# Inflammatory, metabolic, and sex-dependent gene-regulatory dynamics of microglia and macrophages in neonatal hippocampus after hypoxia-ischemia

**DOI:** 10.1016/j.isci.2024.109346

**Published:** 2024-02-28

**Authors:** Elena Di Martino, Anoop Ambikan, Daniel Ramsköld, Takashi Umekawa, Sarantis Giatrellis, Davide Vacondio, Alejandro Lastra Romero, Marta Gómez Galán, Rickard Sandberg, Ulrika Ådén, Volker M. Lauschke, Ujjwal Neogi, Klas Blomgren, Julianna Kele

**Affiliations:** 1 Department of Women’s and Children’s Health, Karolinska Institutet, 17177 Stockholm, Sweden; 2 Department of Biomedical and Clinical Sciences, Linköping University, 58183 Linköping, Sweden; 3 The Systems Virology Lab, Division of Clinical Microbiology, Department of Laboratory Medicine, Karolinska Institutet, 14152 Huddinge, Sweden; 4 Department of Cell and Molecular Biology, Karolinska Institutet, 17165 Stockholm, Sweden; 5 Department of Physiology and Pharmacology, Karolinska Institutet, 17165 Stockholm, Sweden; 6 Dr. Margarete Fischer-Bosch Institute of Clinical Pharmacology, 70376 Stuttgart, Germany; 7 University of Tuebingen, 72074 Tuebingen, Germany; 8 Neonatology, Karolinska University Hospital, Stockholm, Sweden; 9 Pediatric Oncology, Karolinska University Hospital, Stockholm, Sweden; 10 Team Neurovascular Biology and Health, Division of Clinical Immunology, Department of Laboratory Medicine, Karolinska Institutet, 14152 Huddinge, Sweden

**Keywords:** Physiology, Molecular biology, Neuroscience, Immunology, Transcriptomics

## Abstract

Neonatal hypoxia-ischemia (HI) is a major cause of perinatal death and long-term disabilities worldwide. Post-ischemic neuroinflammation plays a pivotal role in HI pathophysiology. In the present study, we investigated the temporal dynamics of microglia (CX3CR1^GFP/+^) and infiltrating macrophages (CCR2^RFP/+^) in the hippocampi of mice subjected to HI at postnatal day 9. Using inflammatory pathway and transcription factor (TF) analyses, we identified a distinct post-ischemic response in CCR2^RFP/+^ cells characterized by differential gene expression in sensome, homeostatic, matrisome, lipid metabolic, and inflammatory molecular signatures. Three days after injury, transcriptomic signatures of CX3CR1^GFP/+^ and CCR2^RFP/+^ cells isolated from hippocampi showed a partial convergence. Interestingly, microglia-specific genes in CX3CR1^GFP/+^ cells showed a sexual dimorphism, where expression returned to control levels in males but not in females during the experimental time frame. These results highlight the importance of further investigations on metabolic rewiring to pave the way for future interventions in asphyxiated neonates.

## Highlights

• Infiltrating macrophages and microglia transcriptomes converge over time
• Macrophages are the main drivers of post-ischemic inflammation
• Sex dimorphism determines the microglia return to homeostasis after HI
• TFs influence lipid/cholesterol and ECM gene regulatory programs during inflammation

## Graphical abstract

**Figure:**
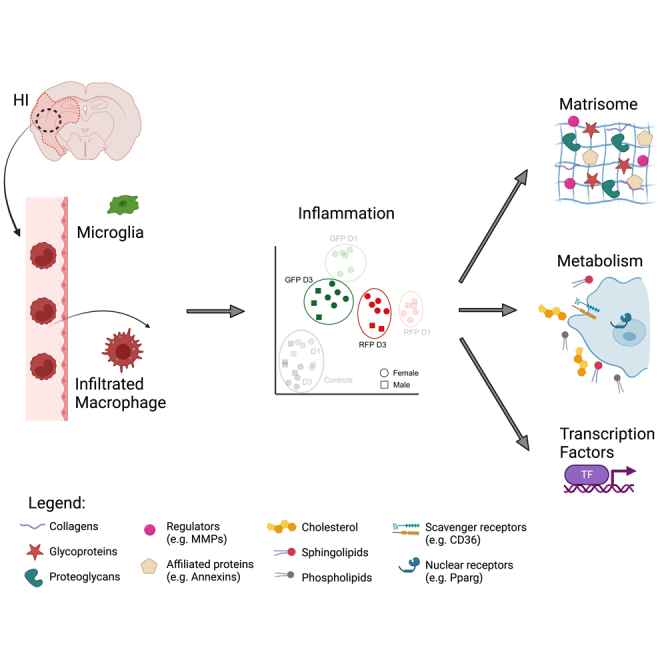

## Introduction

Neonatal hypoxic-ischemic (HI) injury is a complex and severe condition caused by an impaired flow of oxygenated blood to the infant brain and has an incidence of 1–3 cases per 1,000 living births.^1^^,^^2^ To date, therapeutic hypothermia is the only available treatment proven to offer neuroprotection, although about 40% of the treated babies still present long-term disabilities as a consequence of acute neuronal death and chronic neuroinflammation, resulting in neurodevelopmental abnormalities.^3^^,^^4^

It is well established that the rapid migration of immune cells from the blood to the injury site through a disrupted blood-brain barrier (BBB) plays a key role in the post-ischemic neuroinflammatory cascades.^5^^,^^6^^,^^7^ Studies on sterile neuroinflammation, such as perinatal models of brain injury, have shown that peripheral monocytes and macrophages reach the ipsilateral hemisphere as early as 6 h after HI and that their role is fundamental for the progression of the inflammatory response by interacting with brain-resident immune cells.^8^^,^^9^^,^^10^^,^^11^ Microglia are the primary immune cells in the brain. They represent 12%–15% of all cells in the central nervous system (CNS)^12^ and differ from other brain cells by origin, function, morphology, and gene expression patterns.^13^^,^^14^ Contrarily, monocytes derive from bone marrow hematopoietic stem cells, which differentiate into common myeloid progenitors,^15^ and are recruited from the circulation into the CNS to participate in the inflammatory response.^8^^,^^10^^,^^16^ Both macrophages and microglia are sentinels in their specific environment and have phagocytic functions.^17^ They have the capacity to recognize injured cells and immune mediators, to produce signaling molecules and modulate inflammation.^18^ As neuroinflammation is critical for the delayed cell death and the progression of HI brain injury,^19^ understanding the inflammatory dynamics between resident and infiltrating immune cells is paramount to developing future therapeutic approaches. Normal brain development and function requires proper lipid/cholesterol homeostasis, including a period of extensive membrane expansion and myelinogenesis. Present knowledge on lipid/cholesterol dynamics and dysregulation after HI is limited in the immature brain.

Clinical reports and long-term follow-up studies have highlighted that male babies are at higher risk of cerebral palsy and have worse cognitive and motor outcomes after an HI insult compared with females.^20^^,^^21^ The underlying mechanisms behind these sexual dimorphisms are not yet clear. However, it has been suggested that differences in microglial cell density, shape, and function might underlie the increased sensitivity to HI in males,^22^^,^^23^ whereas females are reported to have a more pronounced anti-inflammatory microglial response in chronic stages of HI.^24^ On the other hand, higher rates of macrophage infiltration were observed in the male brain, extending their presence up to 30 days after the injury.^24^ Notably, depletion of myeloid cells was in fact reported to induce neuroprotection only in males.^16^ Although these studies have shed light on sex-dependent differences in the neonatal brain, sex-specific dynamics among resident and peripheral immune cells have not been investigated in the neonatal brain after HI.

In the present study, we investigate the hippocampal temporal and sex-specific gene expression dynamics in both microglia and infiltrating macrophages, one and three days following HI in neonatal mouse pups. For that we apply a modified Vannucci model for neonatal HI in the unique CX3CR1^GFP/+^CCR2^RFP/+^ double transgenic mice. The use of this mouse line allows us to discriminate between resident microglia (CX3CR1^GFP/+^) and infiltrating macrophages (CCR2^RFP/+^). Our findings describe the temporal regulatory landscape including DNA binding motifs and corresponding transcription factors, highlighting the inflammatory, matrisome, and metabolic gene response for microglia and infiltrating macrophages observing infiltrating cells partially converge to a resident immune cell phenotype over time, and that microglial rapid return to homeostasis in males might affect their long-term inflammatory response.

## Results

### Resident microglia and blood-derived macrophages respond differently to HI

**Figure 1:**
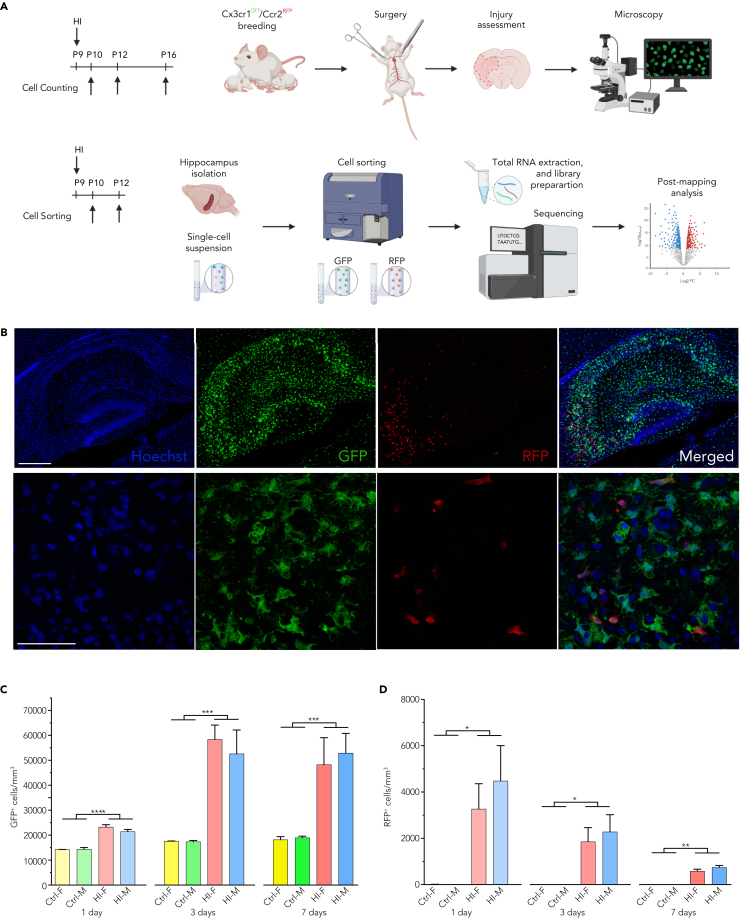
Quantification of resident microglia and infiltrating macrophages in the hippocampus after HI (A) Project design for cell quantification and transcriptomic analysis (created with BioRender.com). (B) Infiltrating macrophages (RFP) and microglia (GFP) in immunofluorescence staining, with scale bar, 250 μm upper quadrant and 50 μm lower quadrant. (C and D) Quantification of CX3CR1^GFP+^ cell (C) and CCR2^RFP+^ cells (D) in the hippocampus after HI (1 day: n = 6 females, n = 8 males; 3 days: n = 7 females, n = 6 males; 7 days: n = 3 females, n = 4 males). Data are analyzed with two-way ANOVA and Šidák-corrected post-hoc t test and presented as mean ± SEM and ∗p < 0.05, ∗∗p < 0.01, ∗∗∗p < 0.001, and ∗∗∗∗p < 0.0001. Legend: control = Ctrl, injured = HI, male = M, female = F. See also Figure S1.

Hypoxia-ischemia-triggered neuroinflammation is characterized by activating resident microglia and infiltrating macrophages. To investigate differences between these two cell types in response to HI, P9 pups of both sexes were sacrificed at day 1, 3, or 7 post-HI for immunohistochemistry quantifications and for transcriptomic analysis (Figure 1A).

Mice had similar body weights at the time of injury (Figure S1A), whereas at the time of sacrifice injured pups were lighter than shams (Figure S1B). There were no differences in axillary temperature in neonatal mice subjected to HI (Figure S1C) and pups with similar neuropathological score (p > 0.05 for all comparisons) (Figure S1D) calculated on Nissl-stained tissues (Figure S1E). These conditions were selected to investigate molecular differences between different time points and between male and female pups.

We validated the transgenic animal model by Tmem119 immunofluorescence (Figure S1F) and quantified CX3CR1^GFP+^ microglia and CCR2^RFP+^ infiltrating macrophage cell densities in the hippocampi of male and female mice pups (Figure 1B). We did observe a few CCR2^RFP+^ cells that were also CX3CR1^GFP+^ at the injury site, but they were not included in the analysis as they were not relevant to the design of our study.

Compared with age-matched controls, CX3CR1^GFP/+^ cells were significantly increased in injured mice at all time points (1 day p < 0.0001; 3 days p < 0.0003; 7 days p < 0.0006), peaking at 3 days after HI in both male and female pups (Figure 1C). There was no significant difference in the number of CX3CR1^GFP/+^ cells between sexes, but tendencies toward higher densities were observed in females compared with males at day 1 after injury (p > 0.05).

No infiltration of CCR2^RFP/+^ cells was observed in control mice, whereas CCR2^RFP/+^ cells were significantly increased in the injured hippocampi at all time points (1 day p < 0.0271; 3 days p < 0.0114; 7 days p < 0.0025), peaking 1 day after HI and slowly decreasing over time (Figure 1D). Moreover, a trend of infiltrating CCR2^RFP/+^ cells was more pronounced in males at day 1 (p > 0.05) and day 3 (p > 0.05) after injury.

Seeing these trends in sex differences in mice with comparable injury, we were curious to further investigate this phenomenon.

### Temporal and cell-type-specific signatures in resident microglia and blood-derived macrophages

Based on the histological outcomes, very few CCR2^RFP+^ cells reached the hippocampi seven days after HI. Thus, the following system biology approach was implemented at day 1 (D1) and day 3 (D3) post-injury to gather further insights into the temporal molecular differences between the two cell types in the hippocampi.

In brief, CD11b^+^/CX3CR1^GFP/+^ cells (“GFP”, resident) were sorted from the ipsilateral hemisphere of injured and control mice, whereas CD11b^+^/CCR2^RFP/+^ cells (“RFP”, infiltrated) were isolated from the ipsilateral hemisphere only in injured mice (Figure S1G).

RNA-sequencing of the sorted cell populations one day and three days after injury revealed a total of 39,501 different coding transcripts.

**Figure 2:**
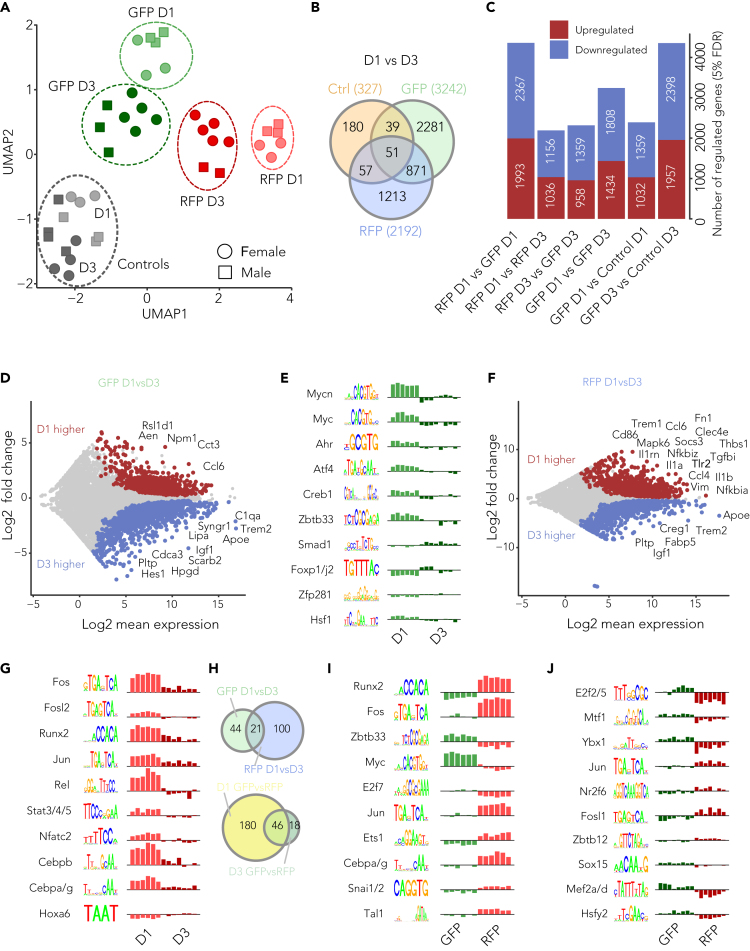
Temporal molecular differences of infiltrating macrophages and resident microglia in the hippocampus (A) Sample distribution based on UMAP dimensionality reduction. Samples in the same group were manually circled. (B) Venn diagram showing unique and commonly regulated (p.adj <0.05, DESeq2) genes between the three pairwise analyses. (C) Bar graph showing number of statistically significant differentially regulated genes (p.adj <0.05) in each pairwise analysis. (D) MA plot visualizes differential gene regulation results of microglia (GFP) day1 vs. day3 post-HI (hypoxia-ischemia). Red and blue dots denote more (p.adj <0.05, DESeq2) and less (p.adj <0.05, DESeq2) expressed genes, respectively, in microglia day1 compared with day3 post-HI. (E) Temporal differences applying the ISMARA algorithm identifying transcription factor binding motifs in microglia. (F) MA plot visualizes differential gene regulation results of macrophages (RFP) day1 vs. day3 post-HI. Red and blue dots denote upregulated (p.adj <0.05, DESeq2) and downregulated (p.adj <0.05, DESeq2) genes, respectively, in macrophages day1 compared with day3. (G) Temporal differences applying the ISMARA algorithm identifying transcription factor binding motifs in microglia. (H) Venn diagram showing regulated (p.adj <0.05, Mann-Whitney U-test with Benjamini-Hochman correction) motifs between cell type and time point. (I) Cell-type-specific differences applying the ISMARA algorithm identifying transcription factor binding motifs 1 day after HI. (J) Cell-type-specific differences applying the ISMARA algorithm identifying transcription factor binding motifs 3 days after HI. Legend: day1 = D1, day3 = D3, control = Ctrl, resident microglia = GFP, infiltrating macrophages = RFP.

HI resulted in rapid and drastic alterations of the microglia transcriptome. Unsupervised clustering analyses were applied to visualize the inter- and intracellular differences in the data. Uninjured controls cluster together as expected. In contrast, GFP samples show two very distinct clusters diverging more at D1 than D3 compared with controls. Similarly, infiltrating macrophages D1 after HI depict higher diversity compared with the other groups. Interestingly, RFP and GFP D3 clusters are remarkably close, suggesting a phenotypic adaptation by infiltrating macrophages after brain entry. Ultimately, macrophage and microglia gene programs tend to converge over time (Figure 2A). The number of differentially expressed genes were compared within and between groups confirming the unique temporal gene pattern for microglia and infiltrating macrophages (Figure 2B).

To better characterize the temporal pattern of these two cells and to understand how their function and roles change during post-ischemic inflammation, we focused on differentially expressed genes (DEGs, q < 0.05) (Figure 2C) and explored the significant motifs regulating the temporal and cell-type-specific differences using the ISMARA algorithm (Figures 2E, 2G–2J).^25^

In GFP samples, various genes involved in inflammatory (*Ccl6*) and apoptotic (*Aen*) signaling and organelle synthesis (*Rsl1d1*) were among the 50 most significantly dysregulated genes D1 after HI. After three days, upregulation of genes involved in cell cycle (*Cdca3*), lipid metabolism (*Apoe*, *Pltp, Hpgd, Lipa*), reorganization of synaptic activity (*C1qa, Syngr1*), or vessel remodeling (*Igf1*) was observed (Figure 2D). In accordance, enriched transcription factor (TF) binding motifs involved in cell cycle (*Max/Mycn*); microglia immune and inflammatory response mediation (*Ahr*); immune cell activation and regulation (*Atf4*, *Creb1*); and stress response, autophagy, and synapse stability modulation (*Hsf1*) were activated D1 post-injury. Also, motifs involved in the regulation of angiogenesis (*Foxp1/j2*) and the TGF-b/Smad1 axes (*Smad1*) were activated D3 after the insult (Figure 2E).

RFP samples revealed an upregulation of inflammatory genes (*Tlr2, Trem2, CD86, Vim*), immune response signaling (*Il1a, Il1b, Ccl4, Ccl6, Socs3*), chemotaxis and cell adhesion (*Fn1, Thbs1, Trem1*), and genes involved in extracellular matrix (ECM) organization (e.g., *Emilin2, Tgfbi*) D1 after injury. Clear elevation of genes involved in lipid regulation and metabolism (*Pltp, Fabp5, Apoe*) was observed D3 after HI (Figure 2F). Enriched TF binding motifs involved in activation of infiltrating cells (*Fos, Jun, Fosl2/Bach2*), induction of cytokines, and activation of other signaling mediators and inflammatory receptors (*Stat4/Stat3/Stat5b, Rela/Rel/Nfkb1, Cebpb, Cebpa/Cebpg*) were induced D1 post-insult. Furthermore, regulation of a TF motif controlling immune cell modulation and cell cycle (*Hoxa6*) was prominent D3 after HI (Figure 2G).

To specifically compare the characteristic features of the two different cell types after HI from an upstream regulatory gene perspective, we focused on the transcription binding motifs that would drive transcription differences between time points and cell types. ISMARA activity analysis showed 180 significant regulators between microglia and infiltrating macrophages D1 post-HI, whereas this had decreased to 18 at D3 (Figure 2H).

Among the top 10 enriched TF binding motifs, *Jun*, known to be involved in myeloid cell activation, was upregulated in RFP samples at both time points. One day after HI, RFP cells show activation of motifs involved in cell matrix regulation (*Etsf1*), cell activation (*Fos*), and monocyte chemotaxis (*Snap/Zeb1/Snai1/2*). In contrast, *E2f7*, which is involved in cell-cycle progression, was suppressed (Figure 2I). Three days post-HI, motifs regulating lipid homeostasis (*Nr2f6)* were active in RFP cells, whereas motifs involved in microglia activation and inflammatory response (*Mef2a/d)* were active in GFP cells (Figure 2J).

In conclusion, infiltrating macrophage differentiation and their altered signal responses are orchestrated by *cis*-linked transcriptional and epigenomic programs that induce gene expression signatures that allow macrophage transition toward an activated microglial phenotype.

### HI modulates the matrisome differently in microglia and blood-derived macrophages

**Figure 3:**
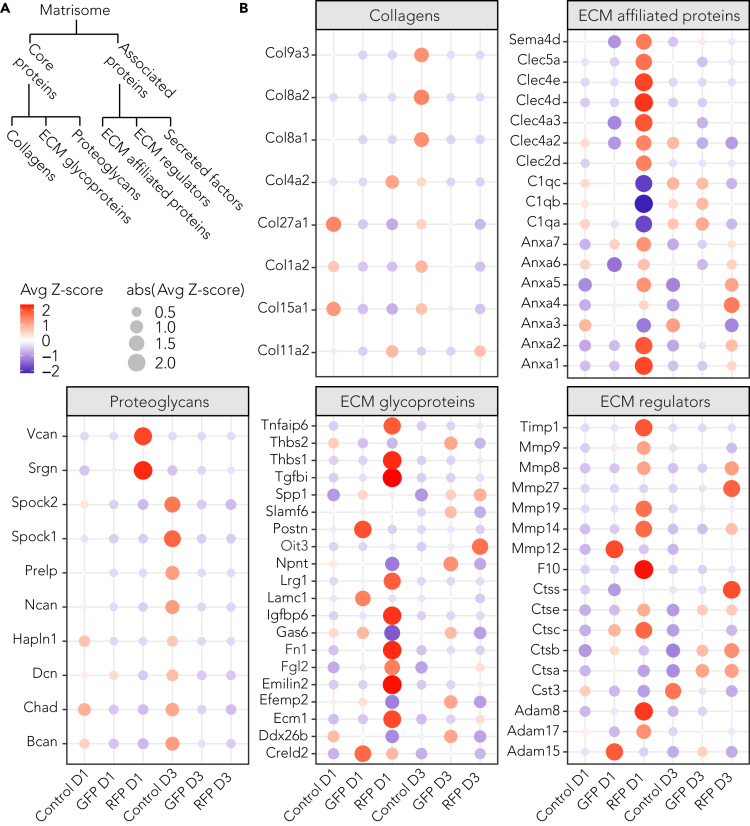
Matrisome gene dynamics in microglia and infiltrating macrophages (A) Matrisome classes. (B) Bubble plots visualizing expression level of genes associated with corresponding matrisome groups (related to Figure 2). RPKM-normalized expression values among the samples were *Z* score scaled, and average *Z* scores per groups are visualized. Bubble size is proportional to the absolute value of *Z* score. Legend: day1 = D1, day3 = D3, resident microglia = GFP, infiltrating macrophages = RFP. See also Figure S2.

Microglia have been implicated in regulating synapse formation and stabilization, as well as promoting synapse plasticity by remodeling the ECM.^8^^,^^26^ To evaluate the gene regulatory patterns that lead to matrix remodeling and readjustment in response to HI, the expression dynamics of matrisome components were assessed by molecular network analysis (Figures 3A and S2).^26^^,^^27^^,^^28^ Notably, most collagens and proteoglycans were downregulated in GFP groups compared with controls. Although expression of ECM glycoproteins was generally elevated in RFP cells D1 after HI (*Emilin2, Ecm1, Fn1, Lrg1, Tnfaip6, Thbs1, Fgl2, Tgfbi, Igfbp6, Creld2*), only a few transcripts were upregulated in RFP samples D3 post-injury (*Oit3, Spp1*). An increased expression of glycoproteins was also observed in GFP groups in the 1-day (*Credl2, Postn, Lamc1*) and 3-day (*Slamf6, Efemp2, Gas6, Npnt, Ddx26, Thbs2*) time points. Analysis of ECM regulators, ECM-affiliated proteins, and secreted factors showed upregulation of transcripts involved in cascades, leading to immune cell activation (*Ctsc, Ctse, Ctss, Adam8, Adam17*), collagen degradation, and tight junction disruption (*Mmp8, Mmp9, Mmp14, Anxa1, Anxa2, Timp1*) primarily in RFP groups. Genes involved in synaptic pruning (*C1q*), and vascular remodeling (*Cst3*) were downregulated in GFP groups (Figures 3B and S2).

Thus, matrisome-associated gene analysis suggests an active role of infiltrating macrophages cells in BBB permeability and immune cells activation. Concomitantly, microglia have reduced ability to maintain BBB integrity and vascular remodeling.

### The temporal dynamics of inflammatory and lipid gene expression differ between cell type

Considering the predominant difference among cells acutely after HI and to further investigate the inflammatory profile of resident microglia and infiltrating macrophages, we conducted pathway analyses D1 after HI.

**Figure 4:**
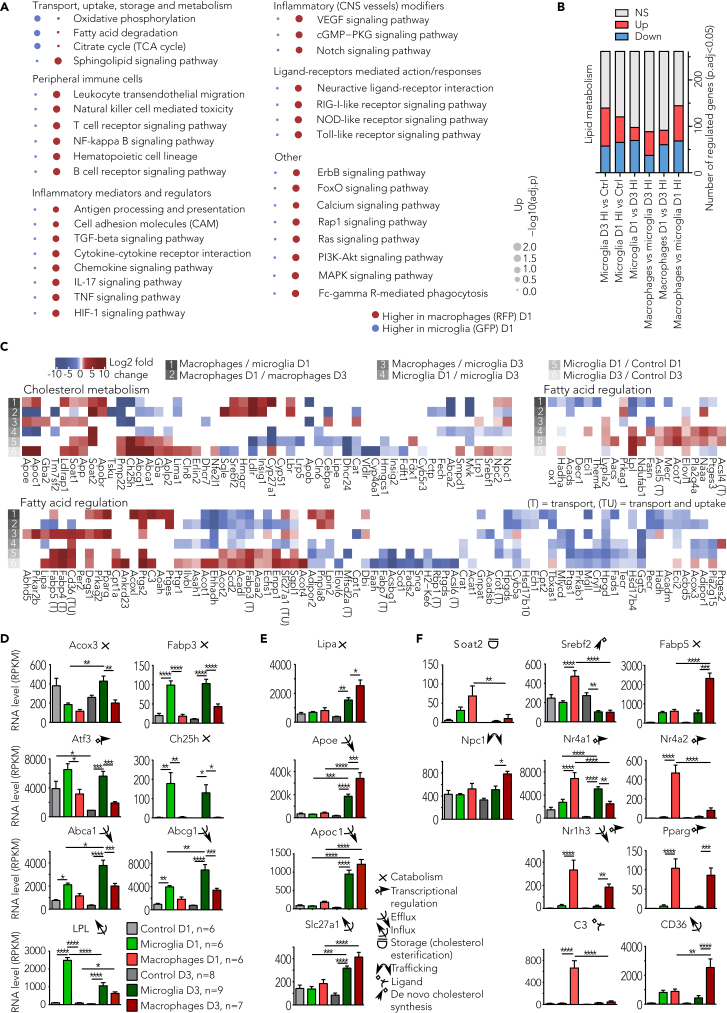
Inflammation and lipid regulation (A) Bubble plot showing the gene set enrichment analysis results of infiltrating macrophages (GFP) vs. resident microglia (RFP) day1 post-HI (hypoxia-ischemia), where red bubbles denote upregulated pathways and blue bubbles denote downregulated pathways in macrophages, and the bubble size is proportional to negative log10 scaled adjusted p values of enrichment (p.adj <0.05, piano GSA). (B) Bar graph representing differentially expressed number of genes in six comparisons. (C) Heatmap showing differential gene regulation status. Log2 scaled fold change values of genes in different pairwise comparisons are visualized, and row annotation represents the differential comparisons. White cells denote non-significant regulation (p.adj >0.05, DESeq2). (D–F) Expression of differentially expressed genes involved in fatty acid and cholesterol processes, with color legend at the bottom of (D) and functional classification at the bottom of (E). The genes are divided by expression pattern into mainly genes expressed in microglia (D), genes also expressed in macrophages (E) or genes expressed mainly in infiltrating macrophages (F). Data are analyzed with one-way ANOVA with Šidák-corrected post-hoc t test and presented as mean ± SEM and ∗p < 0.05, ∗∗p < 0.01, ∗∗∗p < 0.001, and ∗∗∗∗p < 0.0001. See also Figures S3 and S4.

Pathways related to immune cell and sphingolipid signaling were upregulated in RFP cells suggesting that infiltrating macrophages are highly responsive to the post-ischemic microenvironment as soon as they reach the site of injury. GFP cells on the other hand showed significant upregulation in only a few distinct pathways involved in fatty acid degradation, oxidative phosphorylation, and the tricarboxylic acid cycle (TCA) that are associated with microglia activation phenotype (Figure 4A).^29^ These results are in line with the metabolic rewiring of activated microglia/macrophages in response to cytoskeleton rearrangement and production and release of inflammatory molecules.

To further define this outcome, we investigated the gene regulatory pattern of lipid metabolism (Figures 4B and 4C) to assess its correlation with immune cell activation states. We identified downregulation of cholesterol recycling and eliminating genes (*Fdft1, Dhcr24, Hmgcs1* and *Cyp46a1*; Figure S3) and upregulation of cholesterol catabolism genes (*Ch25h*) concurrent to elevated levels of a direct transcriptional regulator of the gene encoding cholesterol-25-hydroxylase that also is a mitigator of microglia inflammatory response (*Atf3*, Figure S4)^30^ in GFP groups, whereas cholesterol efflux transcripts (*Abca1 and Abcg1*) were upregulated primarily in GFP cells D3 after injury (Figures 4C and 4D). Increased expression of genes related to regulation of cholesterol metabolism in macrophages (*Npc1,2* and *Soat1,2*), cholesterol synthesis (*Hmgcr and Srebf1,2*), and cholesterol transport and efflux (LXR1a: *Nr1h3*) was observed in RFP groups at both time points (Figures 4F and S3).

Furthermore, we identified fatty acid metabolism and transport genes to be downregulated in GFP groups compared with controls (Figures 4B and 4C, e.g., *Mfsd2a* and *Adipor1,2*) with a few exceptions (Figures 4D and 4E, e.g., *Lpl, Fabp3, Slc27a1*) and to be upregulated in the RFP (e.g., *Cd36*, *Fabp4, Fabp5, Slc27a1*) group D3 after injury (Figures 4E and 4F). Gene expression involved in lipid uptake (*Pparg*, nuclear receptor) and lipid metabolism (*C3*) was amplified predominantly in RFP cells D1 after HI (Figures 4F and S3). Of interest, expression of nuclear receptors (Nr4A) known to modulate inflammatory and metabolic processes (e.g., via NF-κB *trans*-repression, Figure 4A) were increased primarily D1 post-HI in RFP cells (*Nr4a2*) or GFP cells (*Nr4a1)* (Figures 4F and S3).

In conclusion, the HI-induced neuroinflammatory cascade is accompanied by aberrant lipid metabolism with several forms of fatty acid and cholesterol dysregulation in both cell types. However, after reaching the brain, infiltrating macrophages respond rapidly and appear to be the driving force behind the post-ischemic inflammatory response and distinct cholesterogenic and lipidogenic gene expression patterns. Key regulatory transcription factor(s) and nuclear receptors expressed at the intersection point between lipid/cholesterol metabolic and inflammatory pathways might be attenuators of the neurotoxic inflammatory response after neonatal HI and also serve as important biomarkers and as small molecule targets.^31^^,^^32^^,^^33^

### Cell-type and sex-specific patterns of inflammasome activation

**Figure 5:**
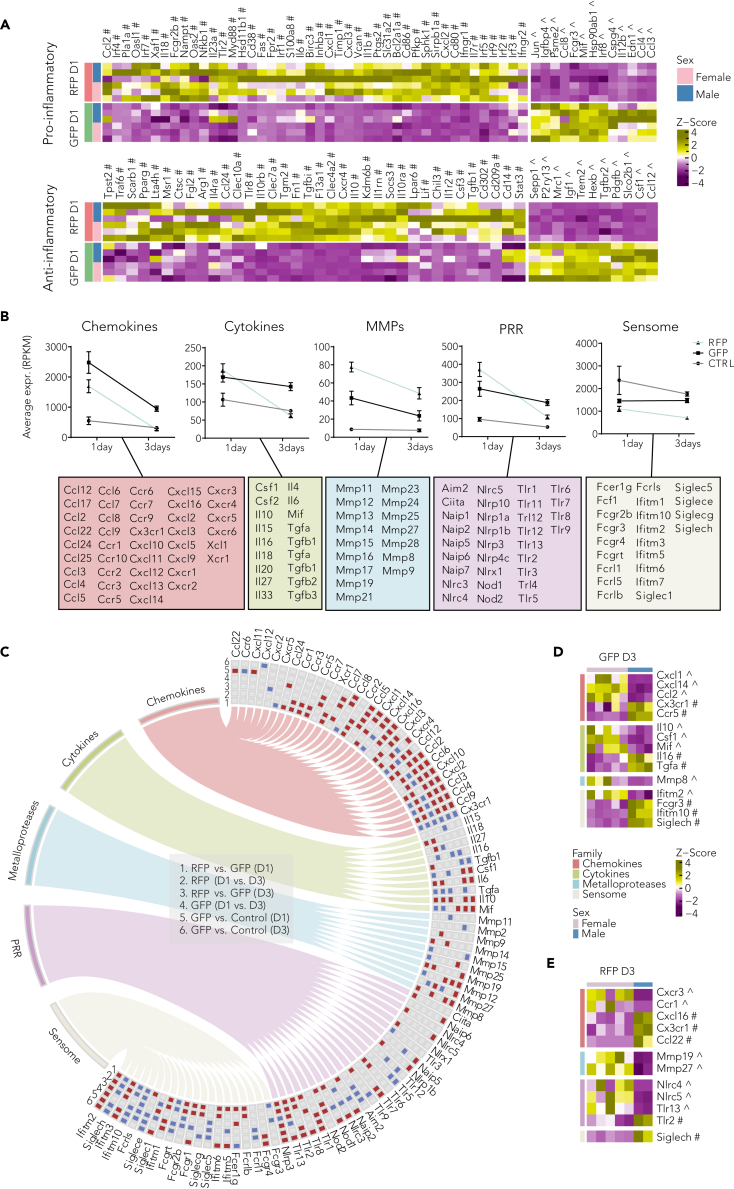
Inflammasome gene regulation depends on post-HI timing, sex, and cell type (A) Expression pattern of significantly expressed (p.adj<0.05, DESeq2) pro-inflammatory and anti-inflammatory subtype markers in microglia (GFP) and infiltrating macrophage (RFP) one day post-HI (hypoxia-ischemia). Heatmap displays Z-transformed RPKM-normalized RNA expression. (B) Average gene expression analysis of top 50 differentially expressed genes (DESeq2 test) comparing resident microglia (GFP), infiltrating macrophages (RFP) groups, and controls (CTRL). (C) Circos plot showing differential regulation status (p.adj<0.05, DESeq2) of genes belonging to sensomes, PRR (pattern recognition receptor), metalloproteases, cytokines, and chemokines families in each of the six pairwise comparative analysis. Ribbons represent association of genes with corresponding gene families. Red color tile denotes upregulation, and blue color tile denotes downregulation of the first term in relation to the second term of the paired comparison. (D and E) Expression pattern and gender-wise regulation status of significantly expressed (p.adj<0.05, DESeq2) genes belonging to chemokines, cytokine, metalloproteases, and sensome families in microglia (D) and infiltrating macrophages (E) three days post-HI. Legend: day1 = D1, day3 = D3, control = CTRL, resident microglia = GFP, infiltrating macrophages = RFP. See also Figures S5 and S6.

Fatty acid synthesis, β-oxidation, and oxidative phosphorylation have been reported to characterize pro- and anti-inflammatory states.^34^^,^^35^ To increase our understanding of the neuroinflammatory contribution of the CX3CR1^GFP+^ and CCR2^RFP+^ populations, we selected a panel of pro-inflammatory (105 transcripts) and anti-inflammatory (92 transcripts) markers (Figure S5).^36^ Our results suggest that RFP cells have a prominent role in orchestrating the inflammation as early as D1 after injury, and the expression of both pro- and anti-inflammatory genes is reduced over time to a level comparable with that of the GFP groups. Specific analysis of the activating genes D1 post-HI revealed the upregulation of the majority of transcripts for both pro- and anti-inflammatory states in RFP cells (Figure 5A).

We observed a higher expression of cytokines and chemokines in GFP compared with RFP and control cells, whereas MMPs and pattern recognition receptors (PRRs) genes displayed more pronounced upregulation in RFP compared with GFP groups (Figures 5B and S6).

One day after injury, an upregulation of chemokine ligands that attract monocytes to the injury site and promote differentiation into macrophages (*CCL3, CCL4, CCL8, CCL12, and CXCL14*) was observed in the GFP group. In contrast, RFP cells displayed upregulation of chemokine receptors (*CCR1, CCR2, CCR3, CCR5, CXCR4, XCR1*) and chemokine ligands that attract neutrophils or control migration and adhesion of monocytes (*CXCL1, CXCL2, CXCL3*). Very few cytokines were upregulated at the one- or three-day time points: specifically, D1 after injury the GFP group had elevated expression of *MIF1* and *CSF1*, whereas the RFP group had higher expression of *IL6*, *IL18*, and *IL10* (Figure S6A). Similarly, analysis of PRRs (Figure S6B) and sensome (Figure S6C) components revealed increased gene expression primarily in the RFP samples D1 after HI. These findings suggest that peripheral macrophages are highly receptive to the environment at the injury site soon after insult and orchestrate the inflammatory cascade. In GFP samples, specifically D1 after HI, a clear downregulation of both PRR and other sensome markers was evident (Figure 5C).

Of interest, expression profiles were significantly different between males and females at the three-day time point (Figures 5D and 5E). When examining the genes differentially expressed in the two sexes, we identified significant upregulation of *Cx3cr1* and *Siglech*, considered to be microglia homeostatic genes, in both cell types of males.

In conclusion, this corroborates that infiltrated macrophages are fast responders to microglia signaling, whereas there was a stronger reduction in microglia activation over time in males.

### Microglial homeostasis genes are differentially regulated in males and females

**Figure 6:**
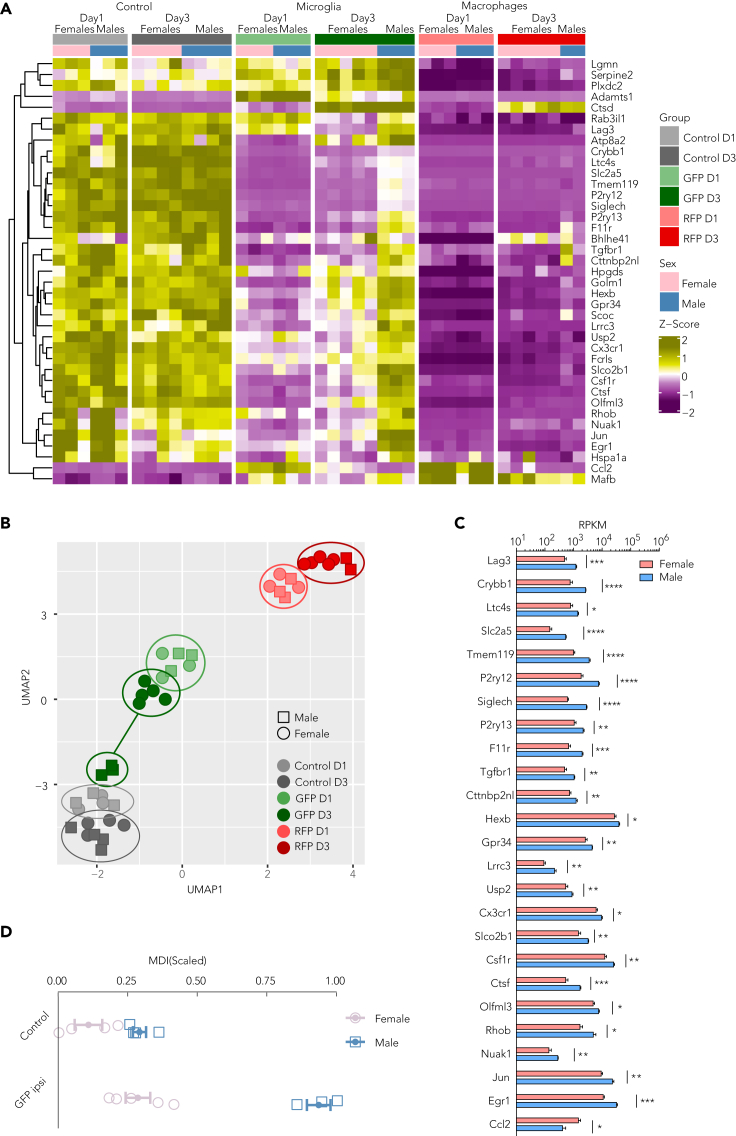
Sexual dimorphism in microglia homeostatic genes (A) Heatmap shows Z-transformed RPKM-normalized RNA expression pattern of microglia homeostatic genes. Column annotation represents sample group and gender information. (B) Sample distribution based on UMAP dimensionality reduction of microglia homeostatic gene expression. (C) Bar plot represents microglial expression day 3 after HI for microglia homeostatic genes that differ significantly between sexes. Data are analyzed with two-tailed unpaired t test and presented as mean ± SEM and ∗p < 0.05, ∗∗p < 0.01, ∗∗∗p < 0.001, and ∗∗∗∗p < 0.0001. (D) Graph showing microglia development index (MDI)^40^ at day 3 with error bars presenting mean ± SEM. Legend: day1 = D1, day3 = D3, resident microglia = GFP, infiltrating macrophages = RFP.

To characterize the temporal dynamics of microglia after HI brain injury, we analyzed a set of microglia-specific markers known as homeostatic genes.^36^^,^^37^^,^^38^^,^^39^ As confirmed by the heatmap (Figure 6A), expression of microglia-homeostatic genes is minimal in RFP samples. One day post-HI, GFP cells showed a clear downregulation of homeostatic genes, as expected after an insult. However, a distinction between male and female samples was observed at D3. Specifically, although female samples showed a prolonged downregulation of homeostatic genes, in males these reverted to control levels displaying sex-specific clustering (Figure 6B). This clear pattern was further confirmed by significant upregulation of 24 homeostatic genes in males (p < 0.05) (Figure 6C).

To determine whether microglia “maturation” was linked to post-injury response, we calculated the microglial developmental index (MDI).^40^ Notably, the analysis confirmed clear differences in GFP MDI between males and females in the injured hemisphere but not between male and female controls (Figure 6D).

Collectively, our findings indicate a sexual dimorphism in microglia that may induce different neuroinflammatory cascades after HI.

## Discussion

Neuroinflammation is a major contributor to neonatal brain injury and is mediated by several different cell types, both local and peripheral, releasing a plethora of signaling molecules upon activation. Microglia and infiltrating macrophages are the main players in the CNS orchestrating the inflammatory cascade after HI and good targets for intervention; however, their signaling with one another and with other brain cells remain poorly understood.

Microglia and macrophages can sense combinations of stimuli and respond according to the microenvironment.^41^^,^^42^^,^^43^ To scrutinize the dynamic nature of these two cell types after a post-ischemic insult, we investigated the transcriptional motif network in combination with the metabolic gene regulatory program and the ECM during the inflammatory response.

The present study meticulously characterizes both cell types over a critical time window, (1) showing that infiltrating macrophages are the drivers of the post-ischemic inflammation, (2) indicating a transcription factor, ECM, and metabolic gene program that partially converges infiltrating cells and activated microglia three days after HI, and (3) suggesting a faster return to homeostatic microglia phenotype in males.

Our findings show a peak of infiltrating CCR2^RFP+^ cells one day post-HI characterized by upregulation of inflammatory genes. Furthermore, the investigation of pro- and anti-inflammatory signaling molecules proposes infiltrating cells having a predominant role in orchestrating the inflammatory cascade. Differences between transcriptomic signatures of CX3CR1^GFP+^ and CCR2^RFP+^ cells diminished over time, suggesting an adaptation of infiltrating macrophages to the brain microenvironment. These results are consistent with other studies showing that peripheral macrophages infiltrating the brain, e.g., after an LPS/HI insult, become “inflammatory microglia”-like cells over time, downregulating CCR2 and upregulating CX3CR1 expression.^44^^,^^45^

**Figure 7:**
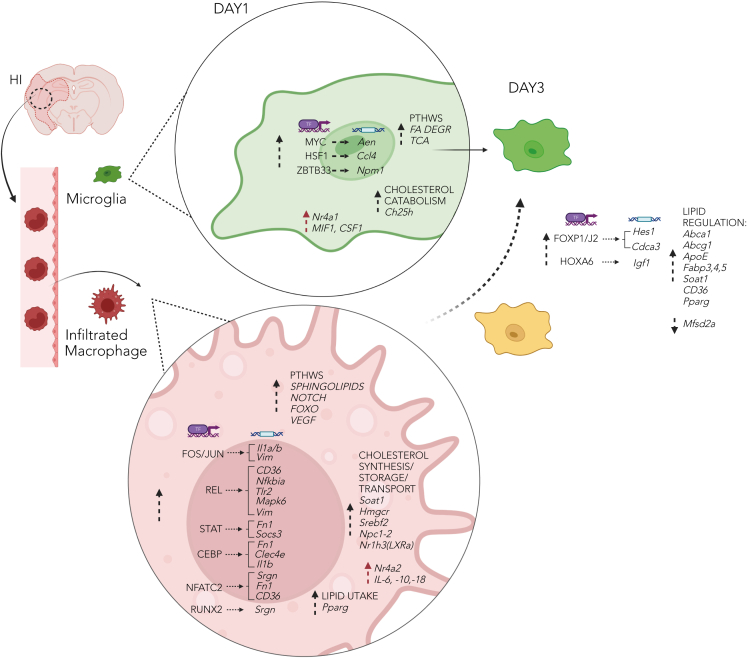
Schematics representing activated microglia and infiltrating macrophages converging over time Upon hypoxia-ischemia (HI), peripheral macrophages are recruited to the injured site by resident cells. Cell-type-specific TF binding motifs and corresponding TFs (transcription factors) regulate inflammatory, ECM (extracellular matrix), and lipid/cholesterol genes during the injury cascade. In microglia: Myc/Max/Mycn (E-box∗) and chromatin remodeler Zbtb33/Chd2 (Kaiso∗) motifs are found near differentially expressed genes Aen and Npm1 (apoptosis and DNA damage response), respectively, Hsf1 modulates the NF-κB pathway, and its motif is found near Ccl4 (inflammation and macrophage chemotaxis). In infiltrating macrophages: Fos/Fosl2/Bach2/Jun and Rela/Rel/Nfkb1 motifs are found near differentially expressed genes vimentin, Il1a/b, and Tlr2 (inflammation and phagocytosis), respectively; Runx2/Bcl11a (Runt∗), Stat4/3/5b, and Cepb (bZIP∗) motifs near Serglycin (*Srgn*) and Fibronectin1 (*Fn1*, ECM remodeling); and Nfatc2 motif near CD36 (regulation of cholesterol and fatty acid transport). Both cell types have upregulation of specific pathways and genes involved in vessels remodeling, leukocyte recruitment, and lipid metabolism one day after injury, although by the 3rd day after HI we observe a convergence in transcript expression. ∗ DNA-binding motif in promoter assessed by ISMARA algorithm. Created with BioRender.com.

The main mechanism of modulation in these highly dynamic cell types occurs via regulatory and chromatin remodeling programs directed by sophisticated TF networks. Our results reveal cell-type-specific transcriptional motifs corresponding to TFs, indicating regulation of our identified inflammatory, matrix remodeling, and lipid/cholesterol gene programs (summarized in Figure 7).

The ECM is dynamically remodeled throughout all phases of cell proliferation, differentiation, migration as well as during injury and inflammation and is now considered to be involved in the neuroinflammatory cascade by directly modulating the expression of cyto- and chemokines.^46^ In the present study, we report the downregulation of context-dependent structural ECM components, such as collagens and proteoglycans, as well as vascular remodeling (*Cst3*) and upregulation of transcripts involved in ECM degradation (*Mmps11/12* and *Adam10/15/33*). Alterations in ECM composition play critical roles in the preservation of BBB integrity as well as in the regulation of the cellular responses that further mediate tissue healing.^47^^,^^48^ Recent findings reported infiltrating macrophages to have an active role in the modulation of the ECM configuration.^49^ In agreement, our data displayed upregulation of several genes giving rise to matrisome or matricellular proteins in infiltrating macrophages involved in BBB permeability (*Anxa1, Mmp9, Timp1*), inflammatory response (*Vcan, Anxa2, s100a10*), and secretion of cytokines (*Srgn*), specifically tumor necrosis factor (TNF), modulating the inflammatory cascade.^50^ Of interest, TNF-α is known to regulate cholesterol by upregulating *Abca1*,^51^ and is also the best-known substrate for *Adam17* elevated one day after injury in CCR^RFP+^ cells, and was recently shown to boost cholesterol efflux in macrophages modulating their immune functions.^52^ Given the bidirectional dialog between microglia/macrophages and ECM, comprehending the matrix in the frame of metabolic rewiring is crucial to pave the way for successful immune-cell-based interventions.^53^

Emerging evidence suggests a link between ECM and lipid metabolism, which regulates multiple biological processes including synaptic formation and axon myelination.^54^^,^^55^ The major component of myelin sheets and CNS cell membranes is cholesterol. Unlike the adult brain, the immature brain has no stable pool of cholesterol, which is instead synthesized *ex novo* and accumulated to comply with the biophysical need of the developing brain.^56^ Currently, this aspect is poorly investigated in neonatal HI. Among the top DEGs, we identified *Lipa*, acknowledged for its role in breaking down lipids such as cholesteryl esters and triglycerides to produce cholesterol and fatty acids, and *Apoe* that facilitates the transfer of cholesterol and phospholipids between cells^34^^,^^57^ and is known to be elevated after injury in neonates.^58^ Accordingly, our data reveal several components of the key regulatory pathways that control lipid metabolism. In resident microglia, there seems to be neither recycling nor elimination of cholesterol (*Fdft1, Lipa, Cyp46a1*) one day after HI but rather an increase in cholesterol catabolism (*Ch25h, oxysterol synthesis*) and efflux (*Abca1*, *Abcg1, Apoe and ApoC1*) primarily at three days post-injury, whereas infiltrating macrophages seem to have *ex novo* cholesterol synthesis (*Hmgcr, Srebf2*) and the consequent efflux regulation (*Nr1h3, Abca1*, *Abcg1 and Apoe*). These infiltrating cells also show increased expression of *Soat1-2*, a significant modifier (cholesterol esterification) of macrophage cholesterol metabolism,^59^ and *Npc1-2*, involved in cholesterol transport and recently reported to influence phagocytic activity.^60^ In accordance, we see increased gene expression of vimentin (*Vim*) and the neonatal glycoprotein *Tnfaip6*. Extracellular vimentin was recently suggested to have a role not only in migration but also in the phagocytosis of activated macrophages in response to TNF-α.^61^

Most of the cholesterol-metabolism-regulating genes we observed are downstream targets of the liver X receptors (LXRs, e.g., *Nr1h3,* an LXRα, found one day post-HI in macrophages). LXRs are activated by oxysterol catabolites in the presence of oxygen, and their expression is reduced during hypoxia and hypoperfusion.^62^^,^^63^ However, upon reperfusion oxysterol production is supposedly restored (as we observe by the upregulation of *Ch25h* in microglia) and the following activation of LXRs results in increased expression of ABC transporters (e.g., *Abca1* and *Abcg1*). Macrophages can modulate the inflammatory response by activating LXRs that are able to *trans*-repress nuclear factor κB (NF-κB) signaling.^64^ Whether microglia have the same or similar ability in the neonatal brain remains to be elucidated; 25-hydroxycholesterol (25-HC), an LXR activator and the catabolite secreted by activated microglia, might modulate lipid metabolism and secretion of ApoE^57^ and ApoC1 by both paracrine and autocrine effects after HI. Pharmacological and genetic modulation of cholesterol esterification enzymes^65^^,^^66^ might be promising targets to evaluate in neonatal HI. Concomitant microglial upregulation of Atf3 (direct regulator of *Ch25h*) might inhibit/dampen the pro-inflammatory (e.g., DAMP/PAMP/TLR) activation as demonstrated in several other disease models e.g., through histone acetylation^67^ or by resolving pro-inflammatory response in activated metabolic rewired macrophages,^68^ but detrimental if lost.^69^ Therefore, Atf3 might serve as a good biomarker and a good candidate for further investigation e.g., as an indirect small molecule target.

One of the debris-clearing outcomes is lipid-laden inflammatory microglia and macrophages with cholesterol as the major component.^70^^,^^71^ Their lipid droplets are generated via the uptake of lipids by scavenger receptors including CD36,^72^ highly expressed after neonatal brain injury^73^ and in accordance with our finding after HI. CD36 is a fatty acid translocase and known to partner with TLR2 during inflammation.^72^ Our observation of increased gene regulation of vimentin accompanied by lipid and inflammatory gene regulation can also be interpreted as a response to counteract CD36 expression and lipid uptake. The intermediate filament version of vimentin not only acts as a key regulator of inflammation in activated macrophages, by the activation of the inflammasome partnering with the MIF-NLRP3 axis,^74^ but also seems to be involved in CD36 regulation affecting inflammation-mediated lipid uptake.^75^

Another fatty acid transporter gene *Mfsd2a*, which we see downregulated in microglia probably as a consequence of hypoxia, and previously demonstrated only in endothelial cells of the BBB,^76^ prohibits the cells from shuttling DHA necessary to counteract the inflammatory responses caused by HI. As a putative mechanism to compensate, we see both microglia and macrophages upregulating genes corresponding to the DHA binding proteins, FABPs, responsible for shuttling the esterified DHA from the plasma membrane (where it is stored) to the organelles for the conversion into pro-resolving lipid mediators to counteract the HI-induced inflammatory response.

Interestingly, DHA is a natural direct ligand of Nr4A2^77^ that is known to modulate inflammatory and metabolic processes.^31^^,^^33^ Moreover, both CD36 and LXRα (*Nr1h3*) are direct targets of PPARγ (*Pparg)*,^78^ which itself is directly regulated by Nr4A1 and has been found as regulator of microglia activation.^79^^,^^80^^,^^81^ In fact, our data display an increase of *Cd36*, *Nr1h3*, *Nr4a1/2*, and *Pparg* primarily in macrophages, suggesting an interplay between these receptors in modulating the cell phenotype (e.g., skewing toward anti-inflammatory macrophages) and the following inflammatory cascade (e.g., regulating the recruitment of macrophages by controlling the production of adhesion molecules or the secretion of chemokines).^82^^,^^83^

While fatty acid synthesis is characteristic for pro-inflammatory cells to promote the formation of pro-inflammatory cytokines, the anti-inflammatory macrophages are characterized by fatty acid oxidation and oxidative phosphorylation.^35^ Our results highlighted the upregulation of metabolic pathways linked to the TCA cycle and oxidative phosphorylation, explicitly in microglia. Previous findings reported that pro-inflammatory microglia and macrophages have an altered TCA cycle; increased levels of citrate; and consequent formation of fatty acids, nitric oxide, and prostaglandins.^84^^,^^85^^,^^86^ In contrast, the anti-inflammatory populations were shown to maintain normal TCA cycle activity and oxidative phosphorylation.^84^^,^^87^ Interestingly, we detected inferred Atf4 activity in microglia. Atf4, a master regulatory TF of the endoplasmic reticulum oxidative stress and autophagy, might be a crucial player of the early metabolic switch in microglial pro-inflammatory phenotype.^88^^,^^89^^,^^90^ This indicates another important checkpoint to take in consideration for modulation in neonatal HI.

In contrast to previous studies that reported that endogenous signals after brain ischemia induced a pro-inflammatory phenotype,^8^^,^^91^ we did not observe that neither pro- nor anti-inflammatory signatures prevailed at any of the time points. We found that the majority of inflammatory markers were upregulated in both cell types at the one-day time point rather than three days post-injury, indicating a quick response. Specifically, microglia exhibited predominant upregulation of chemokine ligands attracting infiltrating macrophages, which in turn overexpressed chemokines receptors, PRRs, and signatures characteristic for sensome. Furthermore, upregulation of the majority of inflammatory transcripts was observed in CCR2^RFP+^ cells, suggesting a leading role of infiltrating macrophages in the post-ischemic inflammation.

Our findings did not indicate any general sex-dependent pattern in pro- or anti-inflammatory signatures; however, we did identify specific genes differently expressed between sexes three days after injury for both CX3CR1^GFP+^ and CCR2^RFP+^ populations as previously reported.^92^,^24^ Among these markers, upregulation of some *bona fide* microglia homeostasis genes (*Cx3cr1* and *Siglech*) was observed in males. Further investigations of homeostatic hallmarks displayed similarities between controls and microglia three days after HI in males. As microglia shift toward an inflammatory phenotype after brain injury, homeostatic genes are downregulated.^36^ Nevertheless, this rapid return to homeostasis in males raises the question about different microglia maturation states among sexes^40^ and the consequent contribution of other cells in the inflammatory cascade.

In the past decade, several studies have described microglial sexual dimorphism concerning cell numbers, morphology, and gene expression in naive animals.^22^^,^^23^^,^^93^ However, we identified no difference in CX3CR1^GFP+^ cell densities between sexes up to seven days after injury. Thus, the recruitment of peripheral immune cells and their contribution in the inflammatory cascade might be the direct consequence of microglia maturation state in the developing brain rather than its number. Previous reports have demonstrated that microglia reach the mature phenotype by the end of the second postnatal week^37^^,^^94^; thus injuries in the immature brain might affect these cells and their gene expression signature. This outcome might imply either an increased signaling from other brain and peripheral cells to complement the lack of microglia activation or a limited response due to a shorter window of resident cell activation.

Taken together, our results provide a comprehensive map of cell-type-specific signature dynamics comprising components of lipid/cholesterol metabolism, PRRs, DAMPs, TREMs, NF-κB signaling, inferred TFs, and nuclear receptors after HI. As such, this study paves the way for the identification of modulators of microglia and macrophage activation, expanding the venue for future metabolic reprogramming of macrophages/microglia in the frame of extracellular matrix and to identify potentially new targets for the treatment of HI.

In summary, the present study comprehensively maps response signatures of resident microglia and infiltrating macrophages in the acute/subacute phase of neonatal hippocampal injury and reveals a sexual dimorphism in the immune response following HI. Specifically, we find distinct DNA motifs associated to transcription factors that modulate genes related to inflammation, sensome, matrisome, and lipid/cholesterol metabolism, and we highlight their response dynamics. These findings present a repository and valuable insights, offering a foundation for further exploration into the specific roles played by microglia and infiltrating macrophages in the inflammatory cascade. This knowledge contributes to identifying the critical time frame for interventions, thereby optimizing therapeutic strategies for neonates affected by asphyxia.

### Limitations of the study

We have not extended the study to evaluate microglia and macrophages in chronic settings after HI due to the limited number of infiltrating cells after seven days. This could be followed-up by single-cell RNA-sequencing. The present study aimed to identify the differences between the two cell types during the acute phase of injury in view of future interventions. However, chronic analysis could be beneficial to detect the signatures of microglia-like macrophages that infiltrated the brain after insult and to evaluate possible variations between males and females. Additional sex differences might be detected with increased number of animals. Although mouse and human neonatal brain have many developmental similarities, it is nevertheless an animal model. Another caveat is the lack of infiltrating macrophages in the absence of insult, precluding a control condition for the infiltrating cells.

Standard differential expression analysis such as ours compares genes’ fractions of the RNA pool rather than absolute amount, which could cause interpretation difficulties upon cell sizes differences between the groups. Also note that the DNA binding motifs in the databases we have applied for our analysis have a non-zero error rate and additionally cannot always pinpoint a specific transcription factor but rather a family of paralogs. The RNA-sequencing protocol we employed did not capture spatial aspects, limiting our ability to study interacting cells.

We identified numerous targets involved in phagocytic function (e.g., autophagy) and cell morphology after HI in the neonatal brain but additional research would be needed to evaluate this specific aspect and investigate phagocytic function in microglia metabolic rewiring in response to HI. Our findings on inferred TF activity in inflammation, matrix, and lipid/cholesterol regulation are some of the crucial denominators as HI proceeds, but further validation of lipid regulating enzymes, metabolic rewiring, modifications/interactions on DNA and protein level, and post-translational modifications at the intersection point of neonatal neuroinflammation would be valuable for the field to follow-up on.

## STAR★Methods

### Key resources table

**Table:** 

REAGENT or RESOURCE	SOURCE	IDENTIFIER
**Antibodies**		

rabbit anti-DsRed polyclonal antibody (1:1000)	Takara Bio	Cat#632496; RRID: AB_10013483
rabbit anti Tmem119 (1:500)	Abcam	Cat#Ab209064; RRID: AB_2800343
rabbit anti ATF3 (1:250)	HPA, Atlas antibodies	Cat#HPA001562; RRID: AB_1078233
goat anti APOE (1:1000)	Millipore	Cat#178479; RRID: AB_10682965
mouse anti RFP (1:500)	Rockland	Cat#200-301-379; RRID: AB_2611063
goat anti GFP (1:2000)	Abcam	Cat#Ab6673; RRID: AB_305643
rabbit anti GFP (1:2000)	Takara Bio	Cat#632592; RRID: AB_2336883
AlexaFlour-488 donkey anti goat IgG (1:1000)	Thermo Fisher Scientific	Cat#A11055; RRID: AB_2534102
AlexaFlour-488 donkey anti rabbit IgG (1:1000)	Molecular probes/Life Technologies	Cat#A21206; RRID: AB_2535792
Alexa Fluor-555- donkey anti rabbit IgG (1:1000)	Molecular probes/Life Technologies	Cat#A31572; RRID: AB_162543
AlexaFlour-555 donkey anti mouse IgG (1:1000)	Biotium	Cat#20037-1; RRID: AB_10854389
AlexaFlour-633 donkey anti rabbit IgG (1:1000)	Biotium	Cat#20125-1; RRID: AB_10853935
CF-633 donkey anti goat IgG (1:1000)	Biotium	Cat#20127-1; RRID: AB_10853302
Brilliant violet 421-conjugated anti-CD11b (1:1000)	BioLegend	Cat#101235; RRID: AB_10897942

**Biological samples**		

Papain 5.0 U/mL	Worthington Biochemical Corporation Roche	Cat# 10108014001
DNase I	Roche	Cat# 10104159001

**Chemicals, peptides, and recombinant proteins**		

Sodium citrate dihydrate (trisodium;2-hydroxypropane-1,2,3-tricarboxylate;dihydrate)	Sigma-Aldrich	Cat# W302600
Hoechst 33342 (2′-(4-Ethoxyphenyl)-6-(4-methylpiperazin-1-yl)-1H,3′H-2,5′-bi-1,3-benzimidazole)	Molecular Probes/Life Technologies	Cat#H3570
Sodium pentobarbital (5-Ethyl-5-(1-methylbutyl)-2,4,6(1H,3H,5H)-pyrimidinetrione)	APL	Cat#338327
Phosphate-buffered saline	Histolab products AB	Cat#BC-PBS940M
Triton X-100 (2-[4-(2,4,4-trimethylpentan-2-yl)phenoxy]ethanol)	Sigma-Aldrich	Cat#T8787
Normal Donkey Serum	Jackson Immuno Research Laboratories	Cat#017000121
ProLong Gold anti-fade reagent	Molecular probes/Life Technologies	Cat#P36930
QIAzol Lysis Reagent	QIAGEN	Cat# 79306
DRAQ5 Fluorescent Probe Solution (5 mM)	ThermoFisher	Cat#62252
Paraformaldehyde (Polyoxymethylene)	Histolab Products AB	Cat# HL96753.1000
Sucrose (β-D-Fructofuranosyl α-D-glucopyranoside)	Sigma-Aldrich	Cat# S7903
Glycerol (propane-1,2,3-triol)	Sigma-Aldrich	Cat# 49781
Ethylene glycol (ethane-1,2-diol)	Sigma-Aldrich	Cat# 03750
Trizma hydrochloride (2-amino-2-(hydroxymethyl)propane-1,3-diol;dihydrochloride)	Sigma-Aldrich	Cat# T3253
Trizma base (2-Amino-2-hydroxymethyl-propane-1,3-diol)	Sigma-Aldrich	Cat# T1503
Sodium Chloride (NaCl)	Sigma-Aldrich	Cat# 31434
Sodium dihydrogen phosphate dihydrate (NaH_2_PO_4_·2H_2_O)	Merck	Cat# 1.06342
di-Sodium hydrogen phosphate dihydrate (Na_2_HPO_4_·2H_2_O)	Merck	Cat# 1.06580
anti-CD11b magnetic beads and columns mouse	Militenyi Biotec	Cat#130-126-725

**Critical commercial assays**		

RNeasy micro kit	QIAGEN	Cat# 74004

**Deposited data**		

Raw RNA.seq data, deposited in European Nucleotide Archive	This study	ENA: PRJEB64216
Processed RNA-seq data, deposited in Gene Expression Omnibus	This study	GEO: GSE238220

**Experimental models: Organisms/strains**		

CX3CR-1GFP knock-in/knock-out mice	Jackson Laboratory	RRID:IMSR_JAX:020940
CCR-2RFP knock-in/knock-out mice	Jackson Laboratory	RRID:IMSR_JAX:004999

**Software and algorithms**		

STAR 2.4.2a	https://github.com/alexdobin/STAR	RRID:SCR_004463
rpkmforgenes	https://github.com/danielramskold/S3_species-specific_sequencing	RRID:SCR_014938
DESeq2 v1.26.0	https://bioconductor.org/packages/release/bioc/html/DESeq2.html	RRID:SCR_015687
ComplexHeatmap v2.2.0	R	RRID:SCR_017270
umap v0.2.6.0	R	N/A
GraphPad Prism6	GraphPad Inc	RRID:SCR_002798
piano	R	RRID:SCR_003200
ggplot2 v3.3.5	R Wickham 2016 https://ggplot2.tidyverse.org	RRID:SCR_014601
ComplexHeatmap v.2.10.0	R	RRID:SCR_017270
CIRCOS v0.69-8 toolkit	R	RRID:SCR_011798
InteractiVenn	http://www.interactivenn.net/	N/A
Zen 2012 SP5	Carl Zeiss	RRID:SCR_018163
StereoInvestigator	MicroBrightField Inc.	RRID:SCR_002526
Code used for the study, deposited in Mendeley Data	This study	Mendeley Data: https://doi.org/10.17632/tnfxthz5nd.1

### Resource availability

#### Lead contact

Further information and requests for resources and reagents should be directed to and will be fulfilled by the lead contact, Julianna Kele (julianna.kele@ki.se).

#### Materials availability

This study did not generate new unique reagents.

#### Data and code availability

• Raw RNA-seq data have been deposited at European Nucleotide Archive and are publicly available as of the date of publication. Accession numbers are listed in the key resources table.
• Processed RNA-seq data have been deposited at Gene Expression Omnibus and are publicly available as of the date of publication. Accession numbers are listed in the key resources table.
• All original code has been deposited at Mendeley Data and is publicly available as of the date of publication. DOIs are listed in the key resources table.
• Any additional information required to reanalyze the data reported in this paper is available from the lead contact upon request.

### Experimental model and study participant details

#### Animals

All animal experiments were approved by the local Animal Ethics Committee (Stockholms djurförsöksetiska nämnd; Ethical approval no.N249/13) and follow the ARRIVE guidelines.

CX3CR1^GFP/+^CCR2^RFP/+^ double transgenic mice were generated in the following way^10^: CX3CR1^GFP/GFP^ and CCR2^RFP/RFP^ mice purchased from the Jackson Laboratory were crossed and first-generation littermates were used. CX3CR1^GFP/GFP^ and Ccr2^RFP/RFP^ mice are on a C57BL/6 genetic background. *CX*_*3*_*CR-1*^*GFP*^ knock-in/knock-out mice express EGFP in microglia inside of the endogenous *Cx3cr1* locus (RRID:IMSR_JAX:020940) and *CCR-2*^*RFP*^ knock-in/knock-out mice contain RFP at the endogenous *Ccr2* locus (RRID:IMSR_JAX:004999). Male and female P9 pups were subjected to experimental procedures and sacrificed at P10, P12 and P16. The presence of the transgenes in the offspring was confirmed by performing PCR. All mice were kept in a humidity-controlled room with a 12h light-dark cycle. Food and water were available *ad libitum*.

### Method details

#### Induction of HI

Unilateral HI was induced in CX3CR1^GFP/+^CCR2^RFP/+^ pups of both sexes at P9 according to the Vannucci model, with some modification.^10^^,^^95^^,^^96^ Animals were anesthetized with 5% isoflurane for induction and 1.5% isoflurane for maintenance in a mixture of air and oxygen (1:1), and the right common carotid artery was ligated with a 6-0 silk suture (Ethicon Inc.). After the surgical procedure, the wounds were infiltrated with bupivacaine (2.5 mg/mL, Marcain; AstraZeneca) for anesthesia. The animals were returned to the dam for 2 h and placed in a chamber perfused with a humidified gas mixture (10% oxygen in nitrogen) for 50 min at 36°C. Their axillary temperature was measured by the thermal probe in the skin pocket between upper foreleg and chest of the animal for about 30 seconds. Control male and female pups were neither subjected to ligation nor hypoxia.

#### Tissue preparation and cutting

Animals were sacrificed at 1 day (1d; males: n=3 control and n=8 HI; females: n=3 control and n=6 HI), 3 days (3d; males: n=3 control and n=6 HI; females: n=3 control and n=7 HI), 7 days (7d; males; n=3 control and n=4 HI; females: n=3 control and n=4 HI) after HI for histological analysis. Animals were anesthetized with 50mg/kg sodium pentobarbital (APL, Stockholm) and perfusion-fixed with a 4% paraformaldehyde solution in PBS (Histolab products AB, Gothenburg). The brains were immersion-fixed in the same fixative for 24 h at 4°C after perfusion and then soaked overnight in graded concentrations of sucrose solution (10, 20, and 30%) containing 0.1M phosphate buffer, pH 7.5. The brains were cut into 40 μm coronal sections in a series of 10 using a sliding microtome (Leica SM2010R). The sections were stored in a cryoprotection solution containing 25% ethylene glycol and 25% glycerol in 0.1 M PO_4_, at +4°C until staining.

#### Evaluation of brain injury

Brain injury in different regions was evaluated in Nissl-stained sections using a semi quantitative neuropathological scoring system.^97^ The cortical injury was graded from 0 to 4, 0 being no observable injury and 4 confluent infarction encompassing most of the cerebral cortex. The damage in the hippocampus, striatum and thalamus was assessed both with respect to hypotrophy (shrinkage) (0–3) and observable cell injury/infraction (0–3) resulting in a neuropathological score (NPS) for each brain region (0–6). The total score (0–22) was the sum of the scores for all four regions. Female and male mice with similar NPS were selected for quantifications to assess the dynamics of resident microglia and infiltrating macrophages after HI.

#### Immunofluorescence

To characterize CX3CR1^GFP+^ and CCR2^RFP+^ cells using immunohistochemistry,^98^ sections were incubated at room temperature (RT) for 1h in blocking solution containing 3% donkey serum (Jackson Immuno Research) and 0.1% Triton X-100 (Sigma-Aldrich) in TBS 1X. Next, the samples were incubated at 4°C for 24h with primary antibody, rabbit anti-DsRed polyclonal antibody (1:1000; Clontech) in blocking solution. The following days the sections were rinsed for 3x10min in TBS 1X, followed by 2h incubation at RT with secondary antibody, Alexa Fluor 555-conjugated donkey anti-rabbit (1:1000; Molecular Probes/Life Technologies) and Hoechst 33342 (1:1000, Molecular Probes/Life Technologies) in blocking solution. Finally, sections were rinsed for 3x10min in TBS 1X, mounted in 0.1M PO_4_ and cover slipped with ProLong Gold anti-fade reagent (Molecular probes/Life Technologies).

For qualitatively assessment of Tmem119, ApoE and Atf3, samples were rinsed in TBS 1X and upon antigen retrieval incubated in sodium citrate solution (NaCi, 10 mM, pH6.0, Sigma-Aldrich) for 30 min at 80°C. Following TBS wash and 1h at RT in blocking solution, 3% donkey serum (Jackson Immuno Research) and 0.1% Triton X-100 (Sigma) in TBS 1X, sections were incubated with primary antibodies at 4ºC for 24 - 72 h, depending on the antibody. The following primary antibodies were used: rabbit anti Tmem119 1:500 (Abcam); rabbit anti ATF3 1:250 (HPA001562, Atlas antibodies); goat anti APOE 1:1000 (Sigma-Aldrich); mouse anti RFP 1:500 (Rockland); goat anti GFP 1:2000 (Abcam); rabbit anti GFP 1:2000 (Clontech). Sections were then incubated for 2h at RT with appropriate fluorescent secondary antibodies. The following secondary antibodies were used: AlexaFlour-488 donkey anti goat IgG (Molecular probes/Life Technologies; 1:1000); AlexaFlour-488 donkey anti rabbit IgG (Molecular probes/Life Technologies; 1:1000); AlexaFlour-555 donkey anti mouse IgG (Biotium, 1:1000); AlexaFlour-633 donkey anti rabbit IgG (Biotium, 1:1000); CF-633 donkey anti goat IgG (Biotium, 1:1000). Hoechst 33342 (Molecular Probes/Life Technologies) was used as a nuclear counterstain. Sections were mounted onto slides and were coverslipped using ProLong Gold anti-fade reagent (Molecular probes/Life Technologies).

#### Microscopy and cell quantifications

The analysis of resident microglia and blood-derived macrophages was performed by quantifying CX3CR1^GFP+^ and CCR2^RFP+^ cells in the hippocampus. For quantification of immune-positive cells, consecutive 3μm z-stack images of the whole thickness of each section were acquired using a Zeiss LSM700 confocal microscope. A blinded offline analysis was performed using Zen Black Software version 3.8 (Carl Zeiss).and all immuno-positive cells were counted in 3-4 sections per animal spaced 400 μm apart. The cell density calculated on regional volumes according to the Cavalieri principle, with the formula:

V=SA×P×T

where V is the total volume, SA is the sum of area measurements, P is the inverse of the sampling fraction of the region (in this case 1/10, i.e., P=10) and T is the section thickness.

Tmem119, ApoE and Aft3 image representations were acquired in sequential scans performed at 1 μm section intervals using a 40× objective lens and 1 airy pinhole setting.

#### Sample preparation for FACS

Animals were sacrificed 1 day (1d; males: n=3 control and n=3 HI; females: n=3 control and n=3 HI) and 3 days (3d; males: n=4 control and n=3 HI; females: n=4 control and n=5 HI) after HI for FACS analysis. Animals were deeply anesthetized with isoflurane and perfused with cold phosphate-buffered saline (pH 7.4) (Life technologies) and brains were removed. The hippocampi from the ipsilateral and contralateral hemispheres were dissected, separately homogenized, and incubated for 20 min at 37°C in a mixture of papain (5.0 U/mL Worthington Biochemical Corporation, to a final concentration of 100 U/l) and DNase (5.0 U/mL, Sigma-Aldrich, to a final concentration of 10 U/l) in 15 mM HEPES buffer consisting of HBSS, 2mM EDTA, and 0.5% bovine serum albumin. Cells were pipetted up and down 20 times and filtered through a cell strainer (40 μm mesh, FALCON) followed by addition of 10% FBS in PBS with 2mM EDTA to a final volume of 10ml and the strainer covered with parafilm was centrifuged in 50mL tubes at 600 g at 4°C for 10 min. After the supernatant was removed, microglia and macrophages were purified with anti-CD11b magnetic beads and columns (Militenyi Biotec). The purified resident microglia and infiltrating macrophages were incubated with brilliant violet 421-conjugated anti-CD11b antibody (1:1000; BioLegend) on ice for 10min. Samples were washed twice with 0.5% FBS in PBS with 2mM EDTA to remove any traces of remaining antibody followed by centrifugation at 600 g at 4°C for 5min and 10 min. Prior to flow cytometry, cells were recovered in 500 μl of 0.5% FBS in PBS with 2mM EDTA.

#### Sample processing and RNA sequencing

CX3CR1^GFP+^/CD11b^+^ and CCR2^RFP+^/CD11b^+^ cells obtained by FACS using MoFlo™ XPD (Beckman Coulter), were further processed for total RNA (RNeasy Micro Kit, Qiagen) followed by pre-RNA Sequencing procedures. Libraries were prepared using SMARTer Ultra Low Input RNA for Illumina and sequenced on HiSeq 2000.^99^ Reads were aligned using STAR 2.4.2a with default settings^100^ against *Mus musculus* reference genome (assembly mm10, without unplaced contigs). Non-uniquely aligned reads were discarded from the alignment. Reads per kilobase of gene per million mapped reads (RPKM) and read counts were generated using the python script rpkmforgenes (available at https://github.com/danielramskold/S3_species-specific_sequencing)^101^ with settings -readcount -fulltranscript -mRNAnorm -rmnameoverlap -bothendceil and RefSeq gene annotation. Gene level read count data was used to find significantly expressed genes between any pair of groups. Differential gene expression analysis was performed using R/Bioconductor package DESeq2 v1.26.0.^102^ Gender information of the samples was added in the differential expression model as covariate to cancel out any possible variance. Genes with adjusted p-value <0.05 were considered significantly expressed. Heatmaps visualizing gene expression pattern were generated using R package ComplexHeatmap v2.2.0.^103^ Volcano plots were made using R package ggplot2 v3.3.2.^104^ Uniform Manifold Approximation and Projection (UMAP) clustering of the samples was performed using R package umap v0.2.6.0.

### Quantification and statistical analysis

All statistical tests except for the test on RPKM values were carried out using GraphPad Prism6 (GraphPad Inc), and were presented as mean ± SEM. Statistical tests on RPKM values were described above. Two-way ANOVA followed by Šidák-corrected post-hoc t-test was used to analyze the neuropathological score, cell density and body weight. One-way ANOVA followed by Šidák-corrected post-hoc t-test was used to analyze differently expressed metabolism regulating genes. P-values <0.05 were considered statistically significant. Data that used ANOVA were presented as mean ± SEM and ∗p<0.05, ∗∗p<0.01, ∗∗∗p<0.001 and ∗∗∗∗ p<0.0001.

ISMARA (Integrated System for Motif Activity Response Analysis) was applied to determine terms of computationally predicted regulatory sites for transcription factors (TFs).^25^ We converted Ismara Z-scores to P-values using the cumulative distribution function of normal distribution and applied Benjamini-Hochberg adjustment, filtering for motifs at a 5% false discovery rate.

Mann Whitney U test was performed on the ISMARA Motif activity values between experimental groups and Benjamini-Hochberg multiple testing correction was applied, represented as q-values.

Sample distribution was accessed through 2-dimensional visualization of the samples after dimensionality reduction by Uniform Manifold Approximation and Projection (UMAP) using R package UMAP v0.2.7.0. Differential gene expression analysis was performed using R package DESeq2 v1.34.0. Sample gender information was used in the design matrix to adjust for the possible bias may arise. Gene-set enrichment analysis was executed using R package piano v2.10.0 and KEGG pathway gene-set for mouse obtained from enrichr web resource. Gene-ontology gene-sets were downloaded from AmiGO version 2.5.17, GO class was set as “direct” and taxonomy Mus musculus was used.

Bar graphs, bubble plots and MA plots were generated using R package ggplot2 v3.3.5. Heatmaps were created using R package ComplexHeatmap v.2.10.0. Circos plot was made using CIRCOS v0.69-8 toolkit. Venn diagram was created using online tool InteractiVenn.
