# Supplementary material for: Inflammatory, metabolic, and sex-dependent gene-regulatory dynamics of microglia and macrophages in neonatal hippocampus after hypoxia-ischemia
Source: iScience. 2024 Feb 28;27(4):109346. doi: 10.1016/j.isci.2024.109346 (PMC10945260; doi:10.1016/j.isci.2024.109346)
Supplement: Document S1. Figures S1–S6 [file mmc1.pdf]

iScience, Volume *27*

Supplemental information

Inflammatory, metabolic, and sex-dependent gene-regulatory dynamics of microglia and macrophages in neonatal hippocampus after hypoxia-ischemia

Elena Di Martino, Anoop Ambikan, Daniel Ramsköld, Takashi Umekawa, Sarantis Giatrellis, Davide Vacondio, Alejandro Lastra Romero, Marta Gómez Galán, Rickard Sandberg, Ulrika Ådén, Volker M. Lauschke, Ujjwal Neogi, Klas Blomgren, and Julianna Kele

Figure S1

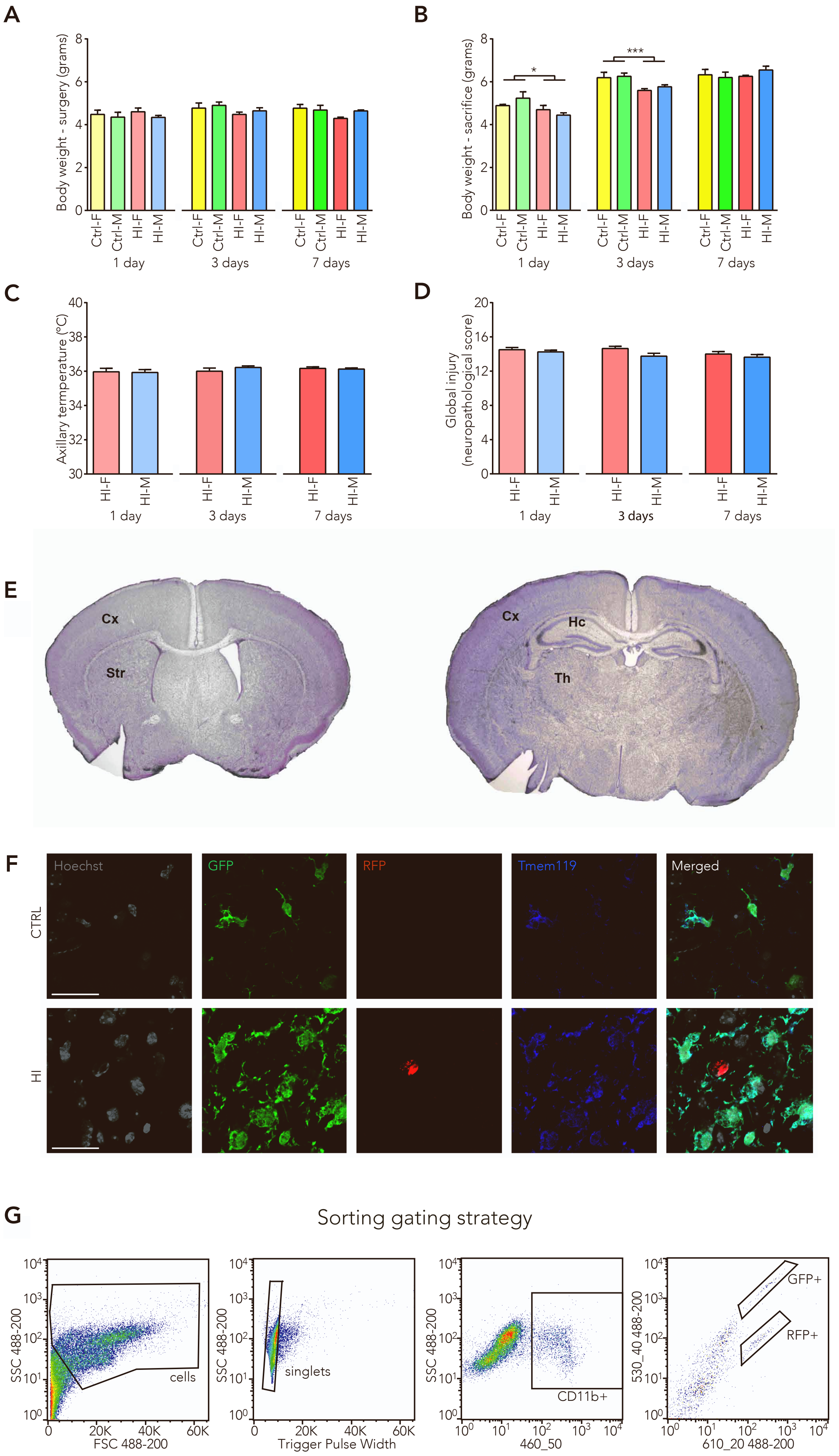
A
Body weight - surgery (grams)
Ctrl-F
Ctrl-M
HI-F
HI-M
1 day
3 days
7 days
B
Body weight - sacrifice (grams)
*
***
C
Axillary termperature (°C)
D
Global injury (neuropathological score)
E
Cx
Str
Cx
Hc
Th
F
Hoechst
GFP
RFP
Tmem119
Merged
CTRL
HI
G
Sorting gating strategy
SSC 488-200
FSC 488-200
cells
Trigger Pulse Width
singlets
460_50
CD11b+
530_40 488-200
610_20 488-200
GFP+
RFP+

Figure S1: General parameters in Vannucci model for neonatal HI in CX3CR1$^{GFP/+}$CCR2$^{RFP/+}$ double transgenic mice, related to Figure 1. (**A-D**) Weight the day of surgery at postnatal day 9 (**A**) and the day of the sacrifice 1, 3 and 7 days later (**B**), axillary temperature (**C**), neuropathological scoring (**D**). (**E**) Nissl-stained sections representing HI injured brains and the anatomical regions assessed for the neuropathological scoring (Cx: cortex, Str: striatum, Hc: hippocampus, Th: thalamus). (**F**) Histological representation of the two distinct cell populations (scale bar is 25 µm) in relation to microglia specific Tmem119 marker. (**G**) FACS gating strategy. In (**A-D**) data are analysed with two-way ANOVA and Šidák-correction post-hoc t-test and no significant statistical difference between animals belonging to the same time point nor between time points was observed ($p>0.05$). Data are presented as mean ± SEM (1 day: n=6 females, n=8 males, 3 days: n=7 females, n=6 males; 7 days: n=3 females, n=4 males). Legend: F=female, M=male, control=Ctrl, hypoxia-ischemia=HI, resident microglia=GFP, infiltrating macrophages=RFP.

Figure S2

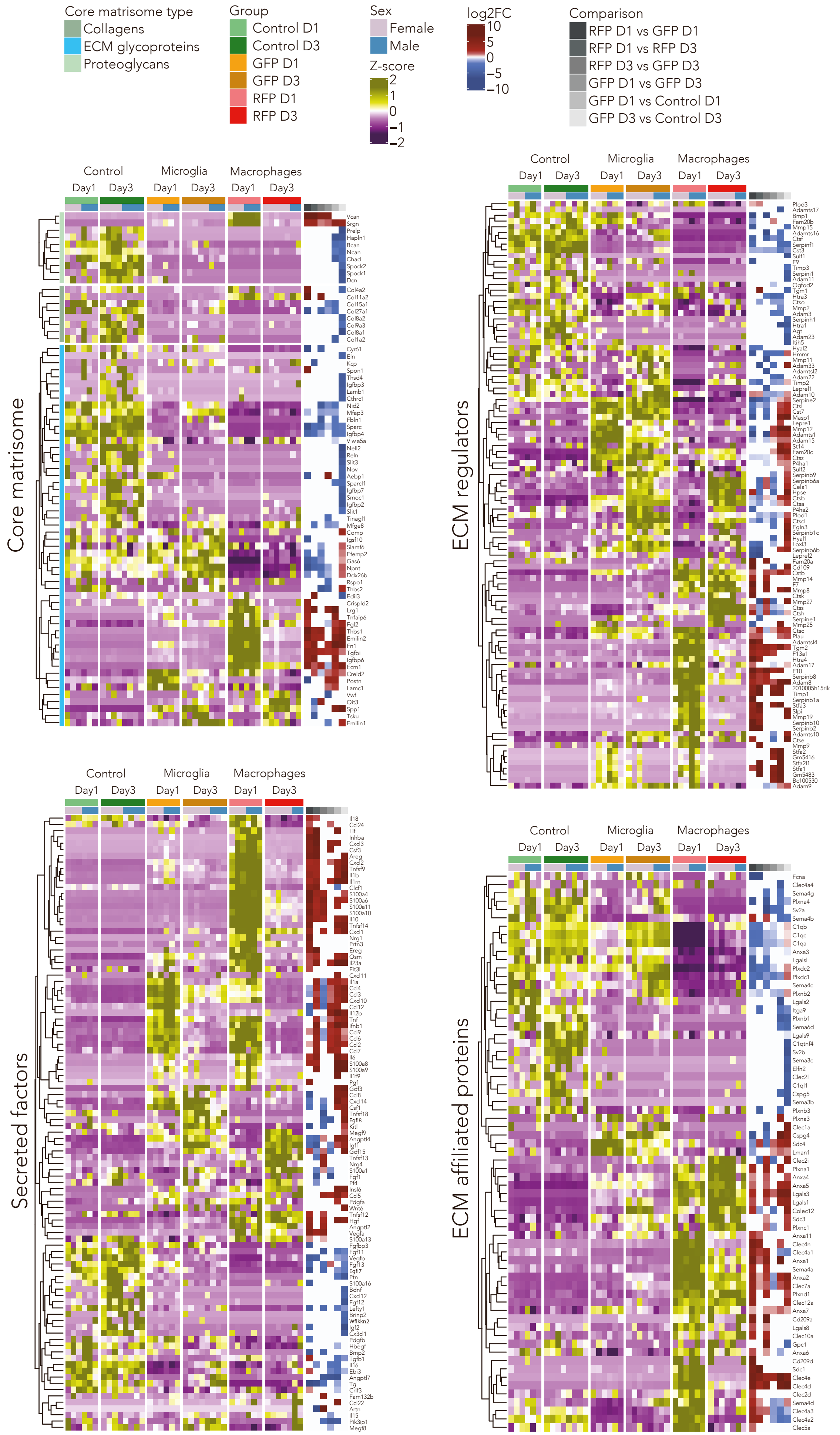

Core matrisome type
Collagens
ECM glycoproteins
Proteoglycans
Group
Control D1
Control D3
GFP D1
GFP D3
RFP D1
RFP D3
Sex
Female
Male
Z-score
2
1
0
−1
−2
log2FC
10
5
0
−5
−10
Comparison
RFP D1 vs GFP D1
RFP D1 vs RFP D3
RFP D3 vs GFP D3
GFP D1 vs GFP D3
GFP D1 vs Control D1
GFP D3 vs Control D3
Control
Microglia
Macrophages
Day1
Day3
Core matrisome
ECM regulators
Secreted factors
ECM affiliated proteins

Figure S2: Expression pattern of matrisome-associated genes, related to Figure 3. Heatmap shows Z-transformed RPKM-normalized RNA expression and fold changes. Column annotation represents sample groups and gender information. Row annotation represent differential regulation status of each gene in each pair-wise analysis (white means adjusted p > 0.05, DESeq2, Benjamini-Hochberg correction) and colour scale reflects the log2 scaled fold change values. Legend: day1=D1, day3=D3, resident microglia=GFP, infiltrating macrophages=RFP.

Figure S3

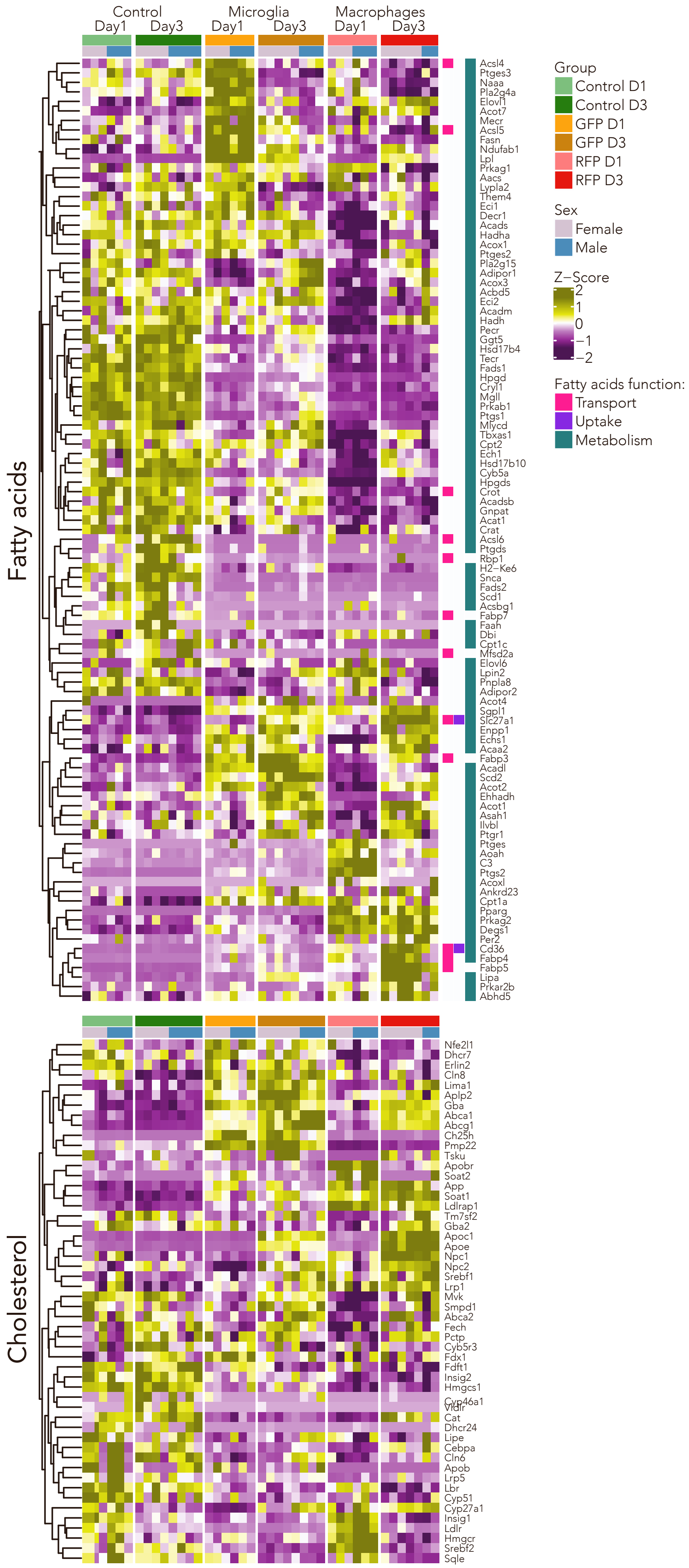

Control
Day1
Day3
Microglia
Day1
Day3
Macrophages
Day1
Day3
Fatty acids
Acsl4
Ptges3
Naaa
Pla2g4a
Elovl1
Acot7
Mecr
Acsl5
Fasn
Ndufab1
Lpl
Prkag1
Aacs
Lypla2
Them4
Eci1
Decr1
Acads
Hadha
Acox1
Ptges2
Pla2g15
Adipor1
Acox3
Acbd5
Eci2
Acadm
Hadh
Pecr
Ggt5
Hsd17b4
Tecr
Fads1
Hpgd
Cryl1
Mgll
Prkab1
Ptgs1
Mlycd
Tbxas1
Cpt2
Ech1
Hsd17b10
Cyb5a
Hpgds
Crot
Acadsb
Gnpat
Acat1
Crat
Acsl6
Ptgds
Rbp1
H2−Ke6
Snca
Fads2
Scd1
Acsbg1
Fabp7
Faah
Dbi
Cpt1c
Mfsd2a
Elovl6
Lpin2
Pnpla8
Adipor2
Acot4
Sgpl1
Slc27a1
Enpp1
Echs1
Acaa2
Fabp3
Acadl
Scd2
Acot2
Ehhadh
Acot1
Asah1
Ilvbl
Ptgr1
Ptges
Aoah
C3
Ptgs2
Acoxl
Ankrd23
Cpt1a
Pparg
Prkag2
Degs1
Per2
Cd36
Fabp4
Fabp5
Lipa
Prkar2b
Abhd5
Cholesterol
Nfe2l1
Dhcr7
Erlin2
Cln8
Lima1
Aplp2
Gba
Abca1
Abcg1
Ch25h
Pmp22
Tsku
Apobr
Soat2
App
Soat1
Ldlrap1
Tm7sf2
Gba2
Apoc1
Apoe
Npc1
Npc2
Srebf1
Lrp1
Mvk
Smpd1
Abca2
Fech
Pctp
Cyb5r3
Fdx1
Fdft1
Insig2
Hmgcs1
Cyp46a1
Vldlr
Cat
Dhcr24
Lipe
Cebpa
Cln6
Apob
Lrp5
Lbr
Cyp51
Cyp27a1
Insig1
Ldlr
Hmgcr
Srebf2
Sqle
Group
Control D1
Control D3
GFP D1
GFP D3
RFP D1
RFP D3
Sex
Female
Male
Z−Score
2
1
0
−1
−2
Fatty acids function:
Transport
Uptake
Metabolism

Figure S3: Expression pattern of fatty acids and cholesterol regulating genes, related to Figure 4. Heatmap shows Z-transformed RPKM-normalized RNA expression. Column annotation represents sample groups and gender information. This lipid metabolism plot is divided into genes regulating fatty acids and genes regulating cholesterol, including transport, uptake and storage subtype indication. Legend: day1=D1, day3=D3, resident microglia=GFP, infiltrating macrophages=RFP.

Figure S4

A

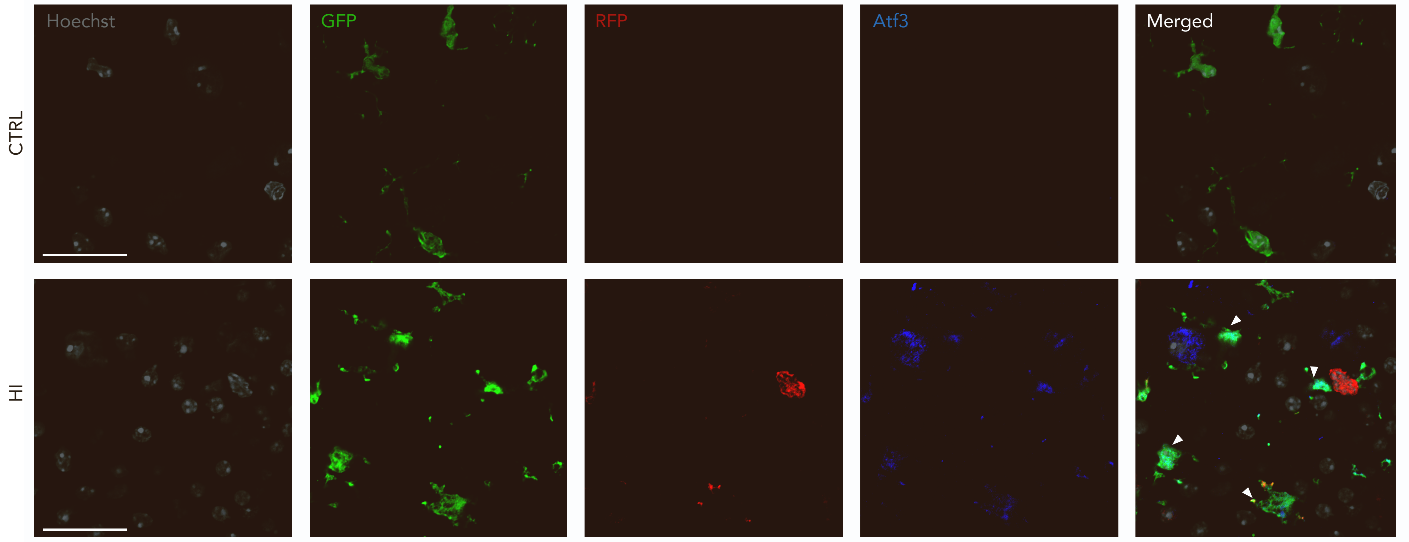

Hoechst
GFP
RFP
Atf3
Merged
CTRL
HI

B

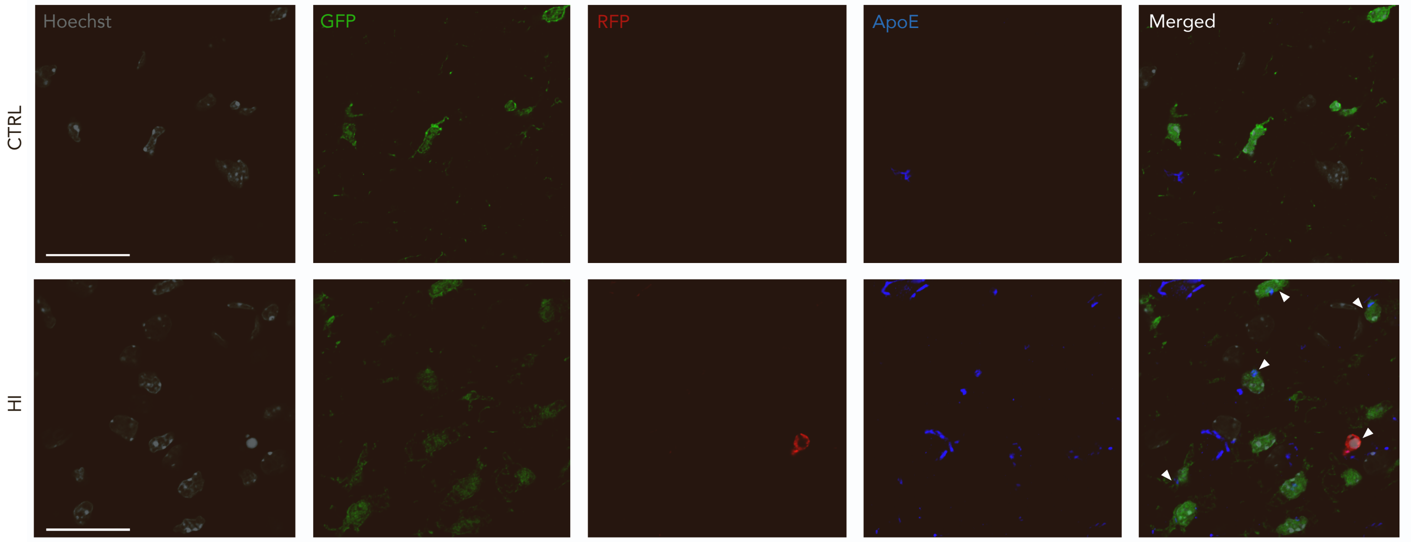

Hoechst
GFP
RFP
ApoE
Merged
CTRL
HI

Figure S4: Qualitative representation of lipid metabolism regulatory markers, related to Figure 4. Histological representation of lipid markers Atf3 (**A**) and ApoE (**B**) in the two distinct cell populations in HI (hypoxia-ischemia) and CTRL (control) samples. Scale bar is 25 µm. Arrows represent colocalization of Atf3 with microglia, and ApoE colocalization with microglia and macrophages. Legend: hypoxia-ischemia=HI; control=CTRL, resident microglia=GFP, infiltrating macrophages=RFP.

Figure S5

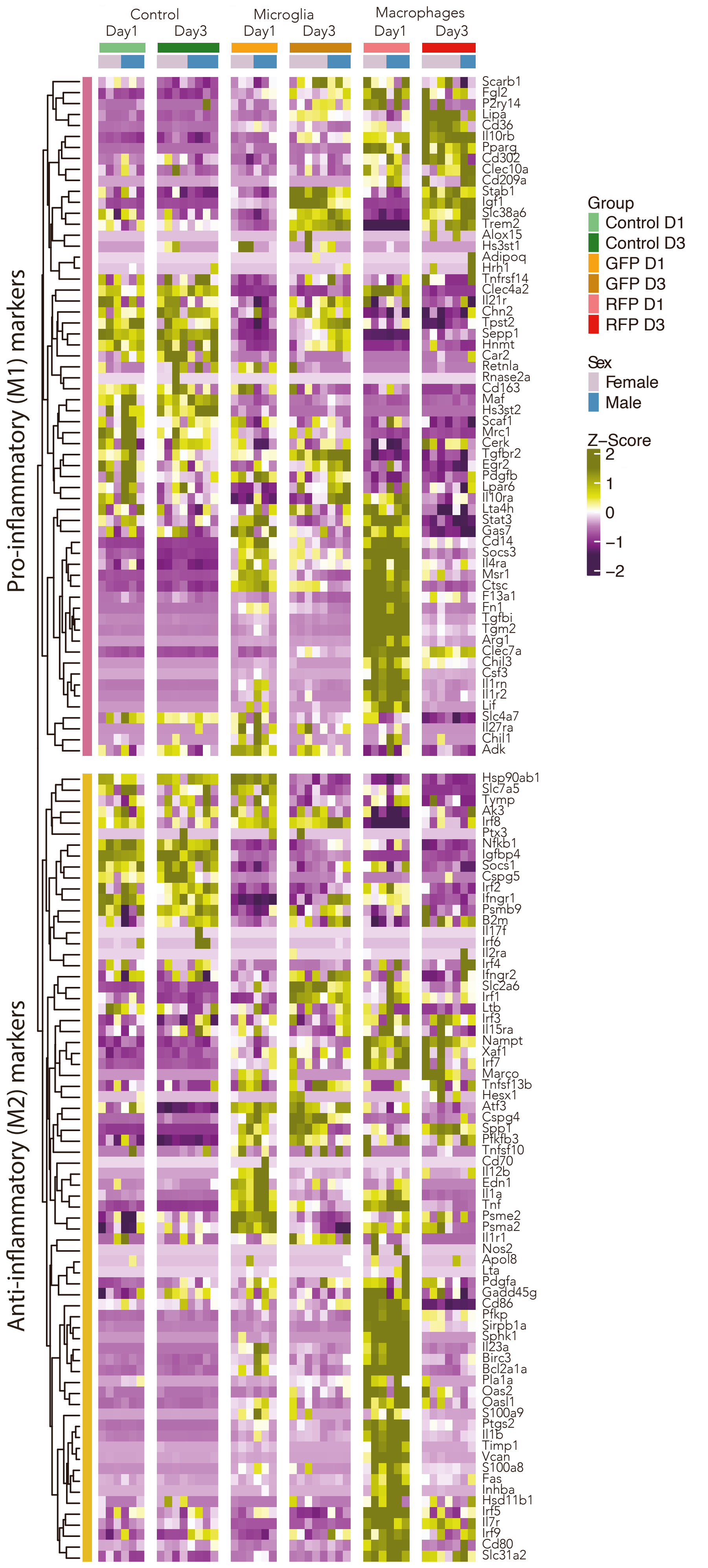

Control
Day1
Day3
Microglia
Day1
Day3
Macrophages
Day1
Day3
Pro-inflammatory (M1) markers
Anti-inflammatory (M2) markers
Scarb1
Fgl2
P2ry14
Lipa
Cd36
Il10rb
Pparg
Cd302
Clec10a
Cd209a
Stab1
Igf1
Slc38a6
Trem2
Alox15
Hs3st1
Adipoq
Hrh1
Tnfrsf14
Clec4a2
Il21r
Chn2
Tpst2
Sepp1
Hnmt
Car2
Retnla
Rnase2a
Cd163
Maf
Hs3st2
Scaf1
Mrc1
Cerk
Tgfbr2
Egr2
Pdgfb
Lpar6
Il10ra
Lta4h
Stat3
Gas7
Cd14
Socs3
Il4ra
Msr1
Ctsc
F13a1
Fn1
Tgfbi
Tgm2
Arg1
Clec7a
Chil3
Csf3
Il1rn
Il1r2
Lif
Slc4a7
Il27ra
Chil1
Adk
Hsp90ab1
Slc7a5
Tymp
Ak3
Irf8
Ptx3
Nfkb1
Igfbp4
Socs1
Cspg5
Irf2
Ifngr1
Psmb9
B2m
Il17f
Irf6
Il2ra
Irf4
Ifngr2
Slc2a6
Irf1
Ltb
Irf3
Il15ra
Nampt
Xaf1
Irf7
Marco
Tnfsf13b
Hesx1
Atf3
Cspg4
Spp1
Pfkfb3
Tnfsf10
Cd70
Il12b
Edn1
Il1a
Tnf
Psme2
Psma2
Il1r1
Nos2
Apol8
Lta
Pdgfa
Gadd45g
Cd86
Pfkp
Sirpb1a
Sphk1
Il23a
Birc3
Bcl2a1a
Pla1a
Oas2
Oasl1
S100a9
Ptgs2
Il1b
Timp1
Vcan
S100a8
Fas
Inhba
Hsd11b1
Irf5
Il7r
Irf9
Cd80
Slc31a2
Group
Control D1
Control D3
GFP D1
GFP D3
RFP D1
RFP D3
Sex
Female
Male
Z–Score
2
1
0
−1
−2

Figure S5: Expression pattern of genes for pro-inflammatory and anti-inflammatory activated microglia/macrophages, related to Figure 5. Heatmap shows Z-transformed RPKM-normalized RNA expression. Column annotation represents sample groups and gender information. Classically activated markers are also annotated as pro-inflammatory and alternatively activated markers as anti-inflammatory. Legend: day1=D1, day3=D3, resident microglia=GFP, infiltrating macrophages=RFP.

Figure S6

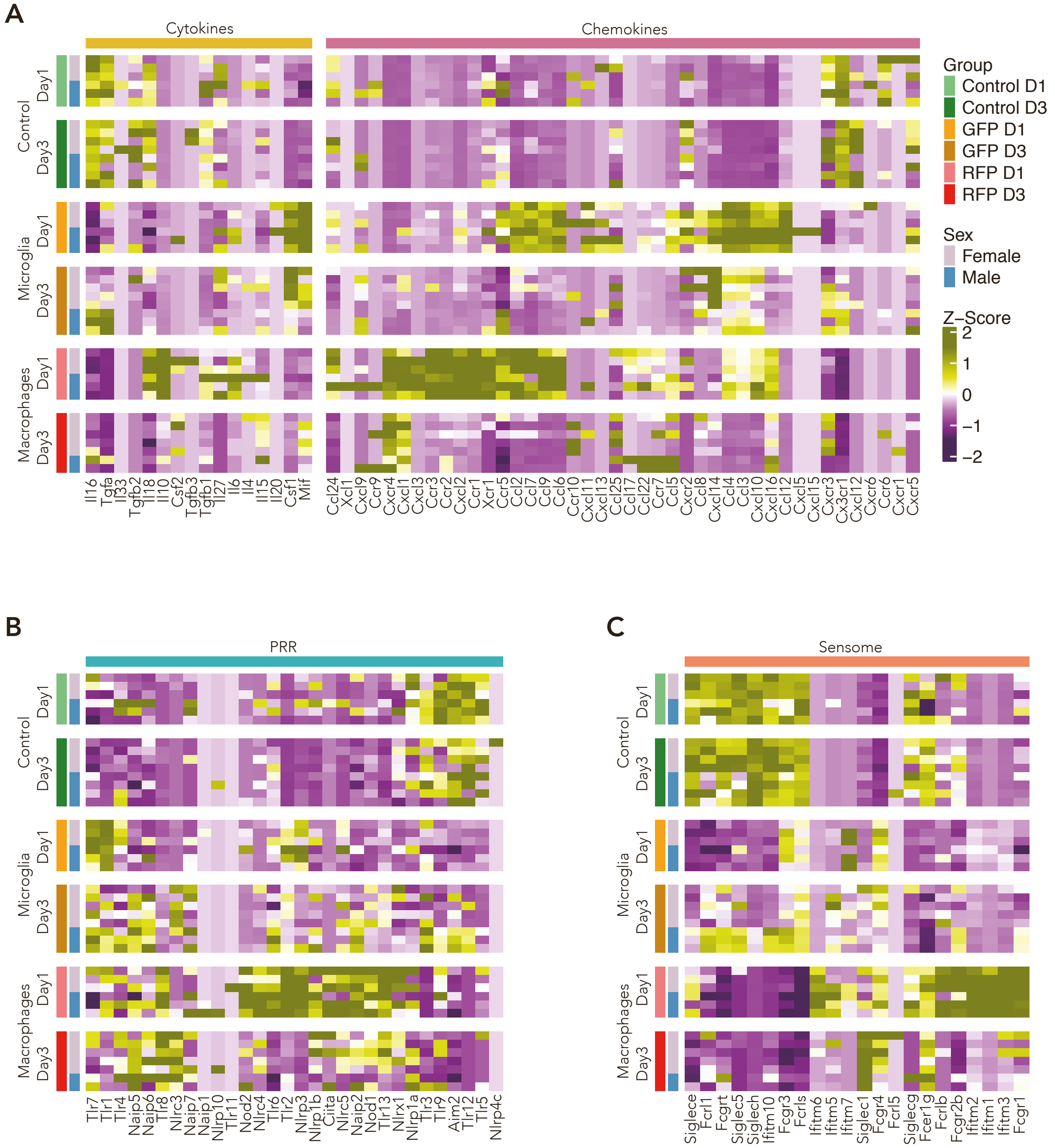
A
Cytokines
Chemokines
Control
Day1
Day3
Microglia
Day1
Day3
Macrophages
Day1
Day3
Il16 Tgfa Il33 Tgfb2 Il18 Il10 Csf2 Tgfb3 Tgfb1 Il27 Il6 Il4 Il15 Il20 Csf1 Mif
Ccl24 Xcl1 Cxcl9 Ccr9 Cxcr4 Cxcl1 Cxcl3 Ccr3 Ccr2 Cxcl2 Ccr1 Xcr1 Ccr5 Ccl2 Ccl7 Ccl9 Ccl6 Ccr10 Cxcl11 Cxcl13 Ccl25 Ccl17 Ccl22 Ccr7 Ccl5 Cxcr2 Ccl8 Cxcl14 Ccl4 Ccl3 Cxcl10 Cxcl16 Ccl12 Cxcl5 Cxcl15 Cxcr3 Cx3cr1 Cxcl12 Cxcr6 Ccr6 Cxcr1 Cxcr5
Group
Control D1
Control D3
GFP D1
GFP D3
RFP D1
RFP D3
Sex
Female
Male
Z–Score
2
1
0
−1
−2
B
PRR
Control
Day1
Day3
Microglia
Day1
Day3
Macrophages
Day1
Day3
Tlr7 Tlr1 Tlr4 Naip5 Naip6 Tlr8 Nlrc3 Naip7 Naip1 Nlrp10 Tlr11 Nod2 Nlrc4 Tlr6 Tlr2 Nlrp3 Nlrp1b Ciita Nlrc5 Naip2 Nod1 Tlr13 Nlrx1 Nlrp1a Tlr3 Tlr9 Aim2 Tlr12 Tlr5 Nlrp4c
C
Sensome
Control
Day1
Day3
Microglia
Day1
Day3
Macrophages
Day1
Day3
Siglece Fcrl1 Fcgrt Siglec5 Siglech Ifitm10 Fcgr3 Fcrls Ifitm6 Ifitm5 Ifitm7 Siglec1 Fcgr4 Fcrl5 Siglecg Fcer1g Fcrlb Fcgr2b Ifitm2 Ifitm1 Ifitm3 Fcgr1

Figure S6: Chemokine, cytokine, PRR and sensome gene expression, related to Figure 5. Heatmap shows Z-transformed RPKM-normalized RNA expression in chemokine and cytokine gene families (**A**), genes belonging to PRR (pattern recognition receptor) (**B**) and sensome (**C**). Row annotation represents sample groups and gender information. Legend: day1=D1, day3=D3, resident microglia=GFP, infiltrating macrophages=RFP.
